# FUNCellA: A Tool for Single-Sample Enrichment Analysis and Relative Pathway Activity Estimation in Single-Cell RNA Sequencing Data

**DOI:** 10.34133/csbj.0053

**Published:** 2026-04-21

**Authors:** Joanna Zyla, Anna Mrukwa, Aleksandra G. Bilska, Kamila Szumala, Joanna Polanska, Michal Marczyk

**Affiliations:** ^1^ Department of Data Science and Engineering, Silesian University of Technology, Gliwice, Poland.; ^2^ Doctoral School of Molecular Medicine, Medical University of Lodz, Lodz, Poland.; ^3^ Computational Biology Lab, Institute of Computer Science of the Polish Academy of Sciences, Warsaw, Poland.; ^4^ Department of Systems Biology and Engineering, Silesian University of Technology, Gliwice, Poland.; ^5^ Breast Medical Oncology, Yale Cancer Center, Yale School of Medicine, New Haven, CT, USA.

## Abstract

Single-cell RNA sequencing (scRNA-Seq) enables advanced analysis of cellular heterogeneity; however, due to data sparsity and noise, analyzing single genes can be challenging. While pathway activity scoring offers a robust alternative to standard single-gene analysis, most available enrichment methods were developed for bulk RNA-Seq or microarray data, so they may fail in addressing the variability and dropout typical of single-cell experiments. Existing approaches primarily focus on comparing pathway activity across predefined groups. Thus, there is a gap in solutions for clustering single-pathway activity vectors, which helps detect subpopulations. Consequently, this limits the functional interpretation and the discovery of meaningful data heterogeneity. We introduce FUNCellA, a framework for estimating relative pathway activity scores using unsupervised tools such as *k*-means and Gaussian mixture modeling. Integrating 7 single-sample enrichment algorithms with novel relative activation thresholding methods, FUNCellA identifies active, inactive, and intermediate cellular states. Benchmarking across scRNA-Seq datasets, both real and simulated one, as well as bulk and microarray transcriptomes, the proposed solution yields outperforming results in relative pathway activation detection compared to existing tools. Finally, FUNCellA enables functional cell classification beyond marker-based clustering, uncovering heterogeneity such as sub-activation states and disease-specific responses.

## Graphical Abstract

**Figure:**
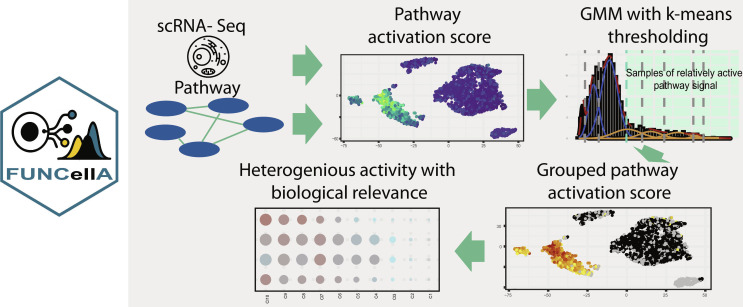

## Introduction

Single-cell RNA sequencing (scRNA-Seq) allows high-resolution profiling of cellular identity and transitions, with gene expression recorded for every single cell [1,2]. Technical dropouts, primarily caused by low mRNA levels or inefficient reverse transcription enzyme activity, can result in a substantially higher number of zeros compared to bulk RNA sequencing [3,4]. These result in noisy and sparse measurements, further compounding the problem of distinguishing biologically meaningful signals from technical artifacts, especially in single-gene analyses [5]. Moreover, a common challenge in the analysis of single-cell data is the lack of reliable cell type annotations, which limits the ability to interpret gene expression patterns in biologically meaningful context. This issue is further intensified by the complexity and heterogeneity of cellular states [6]. In fields such as immunology or oncology, biologically relevant cell states or subpopulations with uncommon cell types [7] are often characterized by the absence of marker genes and subtle activation patterns, rather than clear changes in the expression of a single marker [8]. Thus, analyzing multiple genes simultaneously is a substantial alternative to single-gene-level analysis.

Several methodological strategies have been proposed to represent gene expression in terms of interpretable gene sets rather than individual genes. These include approaches based on feature selection combined with dimensionality reduction [9] as well as methods that construct landmarks variables by merging genes demonstrating similar expression patterns into consensus vectors [10]. Functional analysis reduces the gene expression profiles to a smaller set of biologically relevant features, enabling the derivation of variables that retain clear biological interpretability and functional meaning. During aggregation of gene expression to pathway-level scores, the impact of stochastic noise and increased dropout can be obscured, making underlying signals easier to detect and interpret [8,11]. Pathway activity scores (PASs) are also more directly related to phenotypic impact by providing a functionally relevant simplification of the underlying transcriptional state. Importantly, pathway-level scoring allows the identification of functional cell states masked by traditional clustering methods or markers. For instance, the metabolic reprogramming of tumor-infiltrating immune cells through a glycolytic program is revealed by pathway activation patterns [12]. Since the multimodal and spatial single-cell profiling technologies have become available, PASs represent an additional layer of integration useful to broad analysis of heterogeneous data [13]. Lastly, it might capture the heterogeneity of samples and indicate new response patterns in diseases, e.g., tuberculosis [14]. These examples illustrate the role of pathway scoring in mechanistic understanding and functionally oriented precision medicine. In addition, pathway-level scoring provides a statistically sound, biologically reasonable, and clinically relevant interpretive scheme for single-cell transcriptomic information. It preserves the distributed nature of function and allows for the detection of weak but biologically relevant phenotypes, which creates temporal and layered relations of molecular measurements to biology [15].

Two groups of pathway enrichment methods can be distinguished: (a) differentially expressed-based, which utilize gene-level comparisons between groups to identify dysregulated pathways, and (b) single-sample enrichment algorithms (ssEAs), also known as pathway scoring, where the signal is transformed to PAS for each sample separately. Typically, in single-sample methods, differentially expressed pathways (DEPs) are subsequently inferred based on the previously obtained clusters or phenotypes. The development of ssEA has been closely linked to high-throughput (HT) technologies. For bulk RNA-Seq, GSVA [16] is widely used, whereas single-sample gene set enrichment analysis (ssGSEA) [17] and PLAGE [18] were commonly applied to microarray experiments. For scRNA-Seq, specialized methods like Vision [19] or mitch [20] have been introduced to estimate pathway activity at the individual cell level, taking into account the unique sparsity and variability of single-cell data. A major concern arises when ssEA developed for older HT techniques is applied to single-cell data, as they often do not account for the high variability, sparsity, and technical noise. Some of these limitations have been addressed in large benchmarks [21–23]. Additionally, in [24], the authors compared several ssEA in terms of their ability to support visualization and clustering of scRNA-Seq. Despite these advances, deriving meaningful biological interpretations from clustering results remains a major challenge [25].

Most ssEA studies focus on comparing PAS matrices across pathways and groups, but few examine a single PAS vector, i.e., the activity of one pathway across all [11]. Examining pathway scores at this level can provide valuable insight into cellular heterogeneity. FUNCellA (FUNctional Cell Analysis) aims to identify cells relatively active with respect to a specific signaling pathway or a set of markers. Importantly, it does not rely on any prior grouping; instead, it performs unsupervised clustering solely on pathway activity levels and incorporates activation threshold determination (ATD) to classify cells into active and inactive subpopulations. While there are recent methods that rely on external datasets [26] semi-supervised clustering [27] or resource-expensive gene regulatory modeling [28], FUNCellA offers a simple, fast alternative approach that discovers underlying patterns in the data itself without using prior labels and extensive power. It can be an alternative to many tools that mainly concentrate on annotating cells into known types but do not concentrate on gentle sub-activities [29]. Furthermore, the use of Gaussian mixture models (GMM) allows the detection of intermediate or sub-active states, providing additional insight into cellular heterogeneity, which was never accounted for before. Thus, FUNCellA can help in cell type identification and the characterization of heterogeneity among samples. We tested FUNCellA with 7 ssEA methods, showing their pitfalls and advantages in scRNA-Seq data analysis. FUNCellA is freely available in R and Python (https://github.com/ZAEDPolSl) as well as a web application (https://dssoftware.aei.polsl.pl/FUNCellA/).

## Materials and Methods

### Single-sample enrichment algorithms

In this research, 7 ssEA methods were evaluated. All of them have one common characteristic: The higher the value of the PAS, the more active the pathway is in a particular sample. They can be divided into 2 groups based on the target technology: (a) single-cell sequencing and (b) older HT techniques.

In the first group, the JASMINE [22] algorithm can be distinguished. It ranks the genes for each sample separately and addresses the dropout effect by removing the genes with zero counts for each sample independently. Then, the V statistic is calculated as the mean of the gene ranks in the pathway divided by the number of all nonzero count genes per sample. Next, the V statistic is standardized and averaged with the standardized odds ratio (OR) or likelihood function. The second method, AUCell, was published together with the SCENIC package [11]. Similarly, it ranks the genes for each sample. Further, for ordered gene ranks, it calculates the area under the curve (AUC) for each sample with the first 5% of the ranks to reduce the dropout noise. Next, a new method called BINary Analysis (BINA) was proposed. It is based on evaluating the dropout, defined as the number of nonzero genes per sample in the pathway, and scaling it by the pathway size. This value is defined as drop-ratio (DR). Then, the logit transformation is performed for each DR, as shown in Eq. (1). The constant 0.1 was introduced as an offset to provide numerical stability and prevent undefined mathematical operations in cases of extreme DR values. This parameter is user-adjustable, and the sensitivity analysis (Fig. S1) shows that using a smaller constant increases the derivative of the transformation. This leads to heightened variance for near-zero observations, where minor technical fluctuations in dropout rates are disproportionately amplified into large shifts in the transformed space. The choice of offset = 0.1 suppresses technical noise and stabilizes the log-odds distribution without collapsing the biological signal into a linear scale.

$$\text{BINA}=\log\left(\frac{\mathrm{DR}+0.1}{1-\mathrm{DR}+0.1}\right)$$ (1)

The second group of algorithms was not designed specifically for the scRNA-Seq analysis. The CERNO algorithm [30], next to the *F* statistic, computes the AUC score, the effect size measure used in this research. The genes are ranked per sample (from highest expression to lowest), and the rankings of the genes in the pathway are used to calculate the AUC score based on the Mann–Whitney approach. This method was further applied for scRNA-Seq analysis in [31] as UCell. Next, ssGSEA was tested [17]. The gene ranking is performed for each sample separately, and then the enrichment score (ES) is calculated. The biggest absolute ES is taken as the sample score for pathway activity. The third algorithm is known as the Z-score [32]. The Z-score normalization is performed across the genes, and then it is combined using Stouffer integration [33]. Finally, the average gene signal across the gene set for each sample was calculated in the same manner as in addModuleScore from the Seurat package [34]. Most algorithms were implemented in FUNCellA package from scratch, except AUCell (AUCell package v 1.28.0) and ssGSEA (GSVA package v 2.0.5), for which original implementations are used. JASMINE was updated based on GitHub code to improve computational speed via vectorization while strictly preserving the exact mathematical logic and yielding identical scoring results. Detailed information about running parameters for each of the methods and a condensed summary is presented in Table S1.

### Detection of relative pathway activation

The next step in FUNCellA is to detect samples with relative pathway activation. Here, 2 ATD propositions were introduced and compared to the gold standard solution from the AUCell R package. In UCell package, only manual setting of the threshold is offered. All ATD methods rely on the idea that the value of PAS that is higher than the threshold for a particular sample can be understood as a relatively active pathway. In AUCell, 4 candidate thresholds are calculated. The first one is based on normal distribution parameters and is calculated as *P*(*x* < *T*) ~ *N*(μ_PAS_, σ_PAS_), where *T* is defined as shown in Eq. (2):

$$T=1-\left(\frac{0.01}{\#\text{of samples}}-0.25\right)$$ (2)

**Fig. 1.**
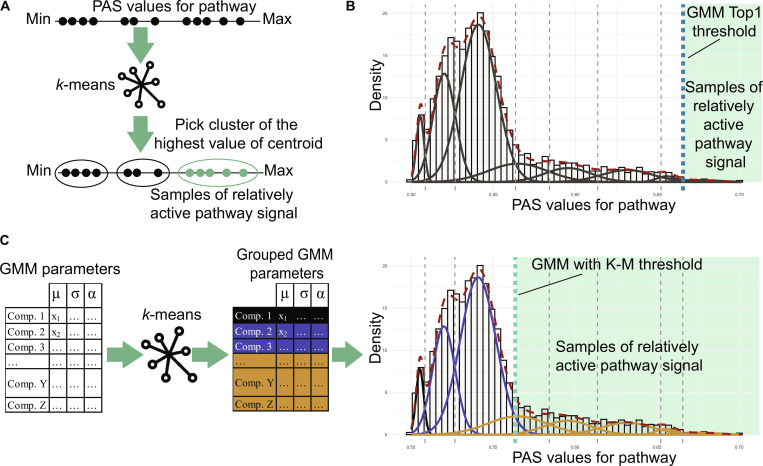
Scheme of the proposed ATD methods. (A to C) K-M, GMM Top1, and GMM with K-M approaches, respectively.

Here, 0.01 (threshold probability) and 0.25 (population percentage) are tunable parameters, although the population percentage correction is omitted for normally distributed data. Next, 2 thresholds are approximated by a GMM decomposition. The authors decompose PAS into 2 and 3 components. For 2-component decomposition, they use Eq. (2) without population percentage correction and *T* = 0.01 for 3 components. To get normal distribution estimates, they use the parameters of the component with the highest mean. The last threshold is estimated as the first minimum after the highest maximum of the kernel density fit. Among 4 thresholds, the highest value is taken. In the FUNCellA package, the original implementation is [11] and will be further named as AUCell thresholding. Additionally, 2 other unsupervised solutions are proposed. The first method applies *k*-means clustering to the PAS vector, with the optimal number of clusters *k* estimated by the Silhouette index. Moreover, multiple starts of the algorithm were implemented to ensure stable determination of initial cluster coordinates (nstart = 25). Samples in the cluster with the highest centroid are considered to have relative pathway activation. This approach is called K-M thresholding. The next proposed method relies on GMM decomposition of the PAS vector into *K* components, where *K* is selected using the information criterion [here Akaike information criterion (AIC)]. The GMM is implemented using the dpGMM R package [35], where initialization is performed via dynamic programming. This GMM implementation ensures a deterministic process of finding model parameters and stable results in threshold determination. Next, the highest threshold from the decomposition is selected. This method is called GMM Top1. As this approach may lead to the selection of only extremely strong activations, the following extension was introduced. The approach named GMM with K-M consists of the following steps: (a) extract the parameters from the GMM decomposition, (b) scale and cluster parameters using *k*-means with selection of *k* according to Silhouette index, (c) group all components belonging to the same cluster, and (d) define threshold as the boundary between the last consecutive component included in cluster and first component from the next cluster. The advantage of the method is the ability to detect sub-activations of pathways in samples. The scheme of all ATD methods is presented in Fig. 1, and their descriptions are provided in Table S2.

### scRNA-Seq datasets and pathway collections

Four publicly available datasets from scRNA-Seq experiments performed on the 10X Chromium Single Cell 3′ v2 platform were used. Each of the analyzed datasets represents different heterogeneous human tissues with annotated cell types.

The first dataset consists of peripheral blood mononuclear cells (PBMCs) from a healthy human 10x_A experiment. The cells constitute a heterogeneous mixture of 9 immunological cell types [36]. The next dataset originates from sequencing bone marrow (BM) cells from a healthy human. This is the most heterogeneous dataset due to the transcriptomic nature of stem cells, in which 14 cell types were identified [37]. Further, the largest set comes from a healthy liver, representing 12 cell types [38]. The last dataset was performed on blood cells from a patient diagnosed with COVID-19. The cells in this experiment were classified into 6 immunological types [39]. The summary of datasets with detailed information is included in Table S3. All datasets were pre-processed on the same manner by filtering out transcripts with low variance of expression across cells using GaMRed [40]. Cells with fewer than 2,500 nonzero gene expression levels were also removed, and each dataset was log-normalized.

A common feature of all datasets is the presence of immunological cells. Among the analyzed datasets, the PBMC was selected as the model dataset in the benchmarking process, as it is the most representative and extensively studied. The remaining datasets were used for validation. Thus, based on PBMC data, the pathways that contain one of the cell types in their name were collected from KEGG [41], CellMarker [42], CIBERSORT [43], PangolaDB [44], and tmod [45,46]. In total, 151 pathways were collected. Next, pathways with low transcript representation in the PBMC dataset (coverage <65%) were excluded, leaving 134 pathways with target cell types listed in Tables S4 and S5.

Finally, to evaluate the performance of the ssEA and ATDs methods in a bias-free, controlled environment, simulated scRNA-Seq datasets were generated using the SPARSim R package [47]. The simulation involved the manipulation of 3 conditions: (a) pathway size, to examine ssEA-mediated noise reduction (*n* equals 15, 20, 25, and 50); (b) fold change (FC), to assess pathway separability (FC equals 1.2, 1.5, 2, 3, 5, and 8); and (c) pathway sparsity, modulated indirectly through FC in SPARSim package. To ensure statistical inference, 5 replicates were produced for each condition, resulting in the generation of 120 datasets. Each dataset consists of 15,000 transcripts across 3,000 cells, with a 4:1 ratio (80% to 20%) between experimental groups. Each ssEA method and ATDs algorithm were run on the simulated datasets. Finally, to check the effect of normalization procedure, the simulated data were pre-processed via simple log_2_ transformation and SCTransform normalization [48,49].

### Benchmarking and evaluation methods

The presented benchmark is mainly dedicated to assess relative pathway activation in samples, but the comparison of an ssEA was also introduced. At first, for the model PBMC dataset, all described ssEA methods were tested for the set of extracted marker pathways with the target cell type (Table S4). Next, for each ssEA, the optimal threshold was determined per tested pathway (threshold step = 0.001) using pathway-specific targets and maximizing the adjusted Rand index (ARI) [50]. Further, a set of pathways with ARI ≥ 0.65 (ARI treated as effect size) was extracted to leave only moderate recovery findings based on research [51]. Following, all 4 different ATD methods were applied for each ssEA and pathways space. Then, for given thresholds, the ARI and false discovery rate (FDR) were calculated using the original cell labels as a reference. To compare thresholding methods, we used the Friedman test [52] with post hoc paired Wilcoxon signed-rank tests, adjusted via the Benjamini–Hochberg procedure [53]. The nonparametric approach was chosen due to the violation of normality assumptions, as confirmed by the Shapiro–Wilk test. In the repeated-measures design, each pathway served as a statistical replicate, with the methods treated as the within-subject factor to account for data dependencies across the identical set of replicates. Further, the Spearman correlation coefficient between the best possible ARI and those obtained from thresholding was calculated. What is more, for BM, COVID, and Liver datasets, the validation was performed using ARI and FDR, and statistical inference was performed in the same manner. The computational time of ssEA was evaluated on 45 exemplary pathways. Calculations were performed on a server running Debian GNU/Linux 11 (bullseye), powered by an Intel Xeon E5-2690 v4 processor (2.60 GHz, 56 cores) and 566 GB of RAM. Two types of timing were recorded: the time required to compute scores for a single pathway across all samples, and the total time to process the full list of 45 pathways. All calculations were run one 1 core to provide information about computational load for regular PC. Finally, the computational load of ATDs was measured on the same set of data. Results of benchmarking were revised on the simulated datasets.

Finally, to show biological relevance of obtained results from GMM with K-M, the exemplary monocyte pathway was selected. At first, the BLUEPRINT resource dedicated for mapping human blood cell epigenomes [54] was used from the celldex package and applied into the popular annotation tool SingleR [55]. Next, to investigate subcluster discrepancies, the differential expression (DE) analysis was performed, using Seurat and DESeq2 testing, between recognized clusters and biological interpretation was introduced.

### Other HT data and validation

The presented approach can be applied to all omics HT data. We collected 3 transcriptomic datasets of breast cancer patients of various histopathological subtypes.

The first dataset comes from a microarray experiment and contains background-corrected, quantile-normalized, and log_2_-transformed expression data from 148 paired breast cancer and normal breast tissues (cohort 2 of E-GEOD-70951) [56]. The second dataset came from The Cancer Genome Atlas (TCGA) breast invasive carcinoma cohort. The RNA-Seq count matrices and the relevant clinical metadata were retrieved from the Genomic Data Commons (GDC) data portal [57,58]. The expression data were normalized using DESeq2 [59] with variance stabilizing transformation. To keep only a homogeneous population, primary tumor samples collected from white females with several available omic experiments were left, excluding formalin-fixed, paraffin-embedded (FFPE) and duplicates, yielding 406 patients [60,61]. The third set of data featured scRNA-Seq profiles from 540 high-quality single-cell transcriptomes [62] [Gene Expression Omnibus (GEO) accession GSE75688]. Samples obtained from the lymph node were removed. Next, the samples were filtered by labels and procedures described in [62], which resulted in 232 samples that were log-normalized. The common breast cancer subtype across all datasets was HER2^+^ (75, 50, and 21 samples out of all for scRNA-Seq, bulk RNA-Seq, and microarrays, respectively) for which signature genes were collected and used as a reference pathway in the benchmarking (Table S6) [61]. Lastly, the scRNA-Seq dataset of much larger volume was collected (GSE182109) [63]. More than 80,000 malignant, neuronal, and immune cells from multiple glioma patients were retained. This dataset was analyzed in the context of the transcriptional markers of glioma-associated microglia and macrophages (GAMs) [64]. The list of the GAMs marker genes is provided in Table S6.

### Implementation

To ensure easy accessibility and provide a robust and reproducible tool for enrichment analysis, we developed open-source software packages for R (FUNCellA) and Python (pyFUNCellA), which are freely available on GitHub (https://github.com/ZAEDPolSl), as well as a website-based application (https://dssoftware.aei.polsl.pl/FUNCellA/). Both packages have identical core functionalities; full documentation and step-by-step running examples are provided. The packages underwent cross-validation for the compliance of results, producing identical results across the platforms. The available application is created with utmost care for the patient’s privacy, with no data storage on the server.

## Results

The evaluation of ssEA methods and thresholding regarding the detection of relative pathway activation can be structured into several different levels: (a) ATD on the model set from the PBMC, (b) ATD validation on other scRNA-Seq datasets, (c) ssEA comparison, (d) biological effects, (e) simulated datasets revision, and (f) other applications on HT data.

### Evaluation of ATD methods for the model dataset

**Fig. 2.**
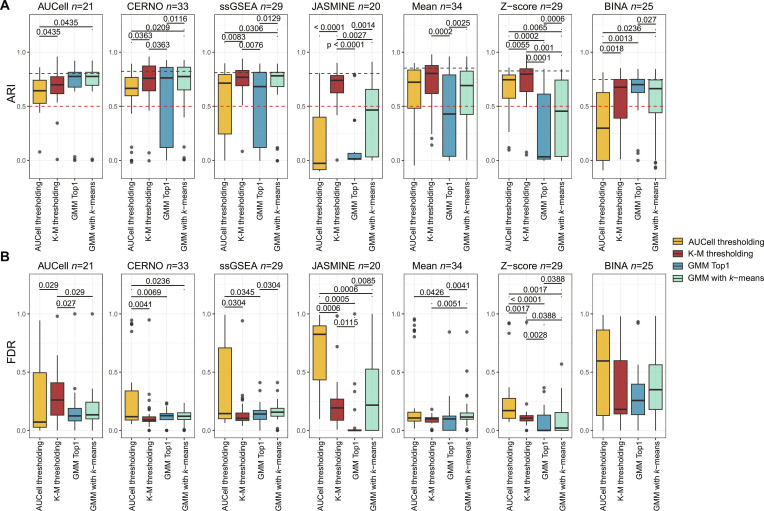
Results of comparison of ATD methods across tested ssEA for model PBMC dataset. On the *Y* axis of (A), the ARI metric result is presented (the higher the better), while on (B) false discovery rate is presented (the lower the better). For both panels, the *X* axis corresponds to the ATD method, which is encoded in color as well. On (A), the red dashed line represents ARI at 0.65 (which corresponds to the moderate recovery), while the gray dashed line represents the median value of ARI for the best possible threshold. The presented *P* values were obtained by pairwise Wilcoxon signed-rank test comparison for paired samples, and only statistically significant outcomes are marked. Next to the ssEA name, the sample size is indicated.

At first, the PBMC dataset was selected as it was extensively studied and often used as a model set. Using the log-normalized counts, the ssEA were run, and pathways with moderate ARI for the optimal threshold were extracted (Fig. S2). As can be observed, out of 134 pathways, the largest number of kept pathways is observed for the Mean (*N* = 34), while the lowest is for JASMINE (*N* = 20). On average, this process reduced the pathways space by ~80%, yet with a strong ability to correctly label relative activation. Moreover, as this process was performed on independent “perfect” fit within each ssEA method and regardless of proposed ATDs, the selection bias was avoided. Next, the Spearman correlation was investigated between ARI obtained for the best possible threshold and the one given by ATD. Figure S3 shows that for rank-based enrichment algorithms (i.e., AUCell, CERNO, ssGSEA, and JASMINE), AUCell thresholding gives the worst results, while GMM with K-M and simple K-M show a strong correlation to the best possible ARI value. For parametric-based ssEA methods (i.e., Mean and Z-score), K-M outperforms other ATD methods. Surprisingly, GMM Top1 shows the best correlation of ARI with the best possible solution for BINA. Subsequently, each ATD technique was compared within ssEA separately. The Friedman test indicated significant differences (*P* < 0.05) between ATD methods for both ARI and FDR. For significant results, the post hoc analysis was performed, and the results are presented in Fig. 2. It can be observed that K-M thresholding, next to AUCell thresholding, is the best ATD method for parametric-based enrichments. For rank-based solutions, K-M thresholding and GMM with K-M give significantly better results compared to AUCell thresholding for CERNO, AUCell, BINA, and ssGSEA, while K-M thresholding was optimal for JASMINE. However, JASMINE overall showed relatively poor performance on real datasets. Finally, the median ARI values obtained by ATD methods were not substantially lower than the median from the best possible thresholds (gray dashed line in Fig. 2A). Moreover, the majority of ssEA methods show the median value of ARI (especially K-M thresholding) higher than 0.65, which represents the moderate recovery (red dashed line in Fig. 2A). This indicates that the ATD approach may have potential in detecting relative pathway activation for individual cells. Lastly, we show results for all 134 pathways regardless of ARI level selection to demonstrate the overall potential of these methods, independent of knowledge about pathway marker ability (Fig. S4). It can be observed that the above observations are consistent when there is no initial pathway selection (ARI ≥ 0.65), proving unbiased benchmarking.

### Validation on a larger dataset collection

**Fig. 3.**
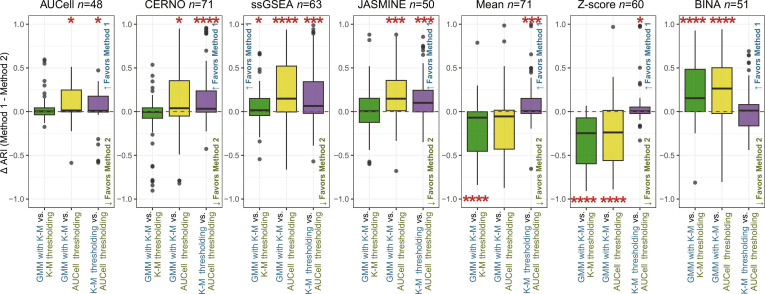
Difference of ARI metric between ATD methods across tested ssEA for the validation set. The *Y* axis represents the difference calculated as Method 1 minus Method 2, which correspond to the first and second algorithms listed in each comparison pair on the *X* axis. As annotated on the plots, positive values favor Method 1, whereas negative values favor Method 2. Statistical significance is denoted as follows: *P < 0.05, **P < 0.005, ***P < 0.001, ****P < 0.0001.

Using the previously selected pathways for each ssEA, the validation process was performed on independent datasets, i.e., BM, Liver, and COVID. Not all cell types observed in PBMC were marked in the validation collection (summary in Table S7). Similarly, within each ssEA, the Friedman test identified significant differences across ATD methods (*P* < 0.05). The post hoc pairwise comparisons are presented in Fig. S5. Most outcomes align with previous findings, but to better illustrate the discrepancies between ATD methods, their pairwise differences are illustrated in Fig. 3 for ARI and in Fig. S6 for FDR. The GMM Top1 algorithm was excluded, as it yielded the poorest results. The figures show that for ssGSEA, GMM with K-M emerges as the best-performing solution compared to other ATDs. In the case of CERNO and AUCell, the K-M method performs as the most effective, following the GMM with K-M (low FDR). For JASMINE, K-M and GMM with K-M perform similarly and better than AUCell thresholding, although JASMINE remains the weakest solution. For parametric-based methods, the K-M is again the best method, and the worst outcomes are observed for GMM with K-M. In addition, GMM with K-M outperforms the rest of ATDs for BINA. Yet, BINA gives poor outcomes in terms of ARI. Furthermore, the high ARI is related to low FDR (as expected), indicating good precision in marking relative pathway activation (Fig. S7).

### Assessment of ssEA methods

**Fig. 4.**
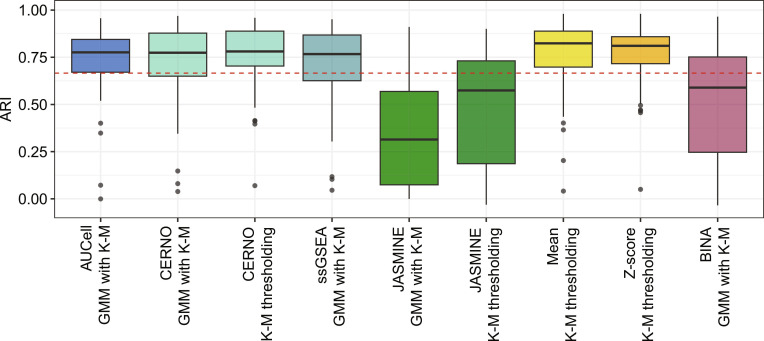
Adjusted Rand index for comparison of tested ssEA methods with their best ATD technique for all common pathways in immunological datasets. The red dashed line represents ARI at 0.65, which corresponds to the moderate recovery effect size used in pathway filtration maximization.

As a next step, for each ssEA, its best ATD method was selected. Then, common pathways across all datasets were extracted, and algorithms were compared to each other (Friedman test; *P* < 0.05). The outcomes of ARI are presented in Fig. 4, FDR in Fig. S8, while *P* values from pairwise comparisons are presented in Table S8. BINA and JASMINE (with both selected ATDs) give significantly worse results compared to the remaining ssEA methods. Yet, BINA combined with GMM with K-M gives statistically better results than JASMINE on K-M. Taking into consideration the rest of the methods, Mean and Z-score combined with K-M are significantly better than ssGSEA with GMM with K-M on ARI and FDR metrics. Next, Mean and Z-score with K-M outperform other solutions in terms of FDR. Yet, for ARI, no differences were observed. Moreover, FDR is sensitive to sample imbalance, and its results should be interpreted with caution.

### Biological discrepancy impact

To better understand the factors influencing method performance, we investigated whether the biological distinctness of cell types affects the accuracy of the ssEA and ATD methods. For this purpose, we quantified the separation between annotated cell types within each dataset using the average Silhouette width and estimated the correlation (Spearman rank correlation) with ARI metric (skipping FDR due to its high correlation with ARI; Fig. S9). As can be observed, the AUCell thresholding is robust to the cell separation in the dataset, regardless of ssEA method when the real biological data are investigated. The remaining ATDs generally exhibit a significant positive correlation between ARI and mean Silhouette width, particularly for CERNO, ssGSEA, and BINA. This indicates that for these combinations, the accuracy of the methods significantly improves when the targeted cell populations are more distinctly separated from others in the dataset. On the contrary, the JASMINE method is largely independent of the overall transcriptomic distance between cell clusters. This phenomenon is also observed for AUCell (except for the GMM Top1 thresholding). Furthermore, it can be observed that results are dependent on the target cell type. For instance, plasmacytoid dendritic cells (purple color on Fig. S9) consistently exhibit the highest separation scores, while T cells frequently display the lowest, indicating a high degree of transcriptomic overlap with other cells in the datasets. Yet, it is well known that T cells divide into smaller, specialized subpopulations (e.g., cytotoxic T cells), which were not considered in the evaluation process and show some limitations of the present benchmark.

**Fig. 5.**
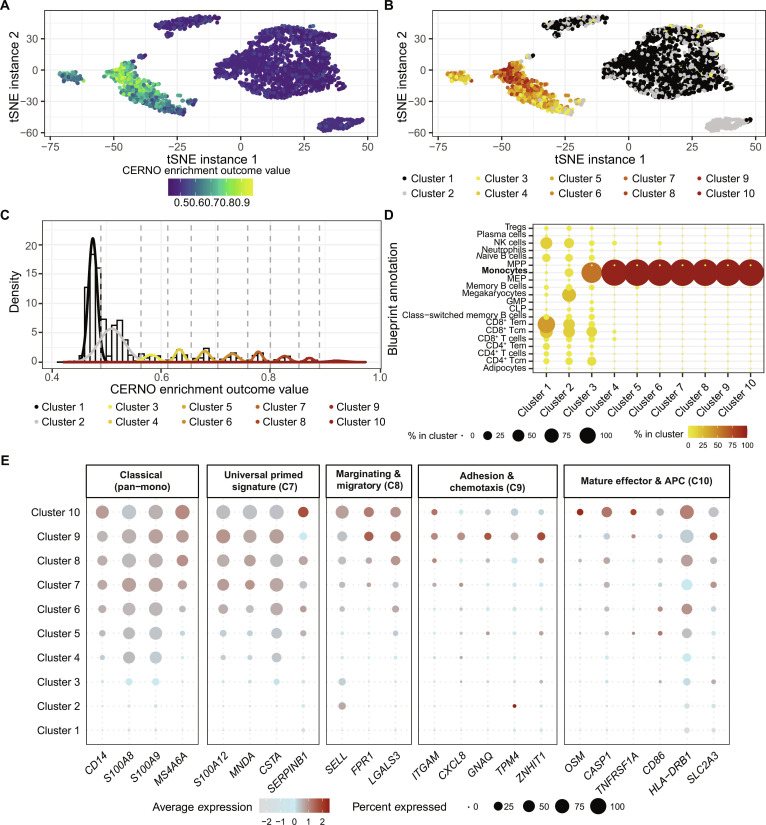
Visualization of results from the CERNO ssEA and GMM with K-M ATD for the monocyte pathway. (A and B) t-Distributed stochastic neighbor embedding (t-SNE) projection of the tested PBMC dataset. (A) Values of PAS obtained by CERNO algorithm. (B) Clustering obtained by GMM with K-M, where intense yellow-red colors show relative pathway activation, and black-gray shows non-active cells for the pathway. (C) GMM that decomposes values from (A), with the presence of active and non-active clusters of the same color-coding as in (B). (D) Cell type annotation of the PBMC dataset based on the Blueprint reference database. Both dot size and color scale indicate the percentage of cells assigned to a specific annotation within each GMM cluster. (E) DotPlot illustrating the average expression and percentage of expressing cells for selected activation marker genes across the GMM clusters. The genes are categorized to highlight the sequential trajectory of monocyte.

Further, to visualize the ability of an ssEA and GMM with K-M, the top 10 monocyte signature from CIBERSORT was extracted. Next, CERNO enrichment combined with GMM with K-M was run, and the result is shown in Fig. 5. The presented visualization is part of the FUNCellA package, and Fig. 5A to C can be obtained for any pathways and ssEA. In Fig. 5A, it can be observed that monocytes are located on the left side of the figure and the PAS values from CERNO are more yellowish. Next, Fig. 5B and C shows the effect of GMM with K-M decomposition and clustering. As can be observed, GMM with K-M decomposition shows different levels of relative pathway activation (clusters), which may help distinguish heterogeneous subtypes. In the next step, the popular annotation method is applied (SingleR). As shown in Fig. 5D, clusters from 3 to 10, which were marked as relatively active by GMM with K-M in BLUEPRINT annotations, are correctly characterized as monocytes. Yet, no distinction in monocytes was recognized. Thus, the DE analysis was performed, using Seurat and DESeq2 testing, between recognized clusters. Results of selected markers are presented in Fig. 5E. At first, we concentrated on monocyte marker genes, i.e., CD14, S100A8, S100A9, and MS4A6A, and grouped them as classical markers (pan-mono) [65,66]. It can be observed that these marker genes are expressed starting from the relatively active cluster 3, confirming the monocyte identity. Next, we noticed that clusters C7 to C10 capture the progressive stages of the inflammatory response: starting from C7, which represents a universal primed signature, through the C8 migration state, next C9 for the adhesion, and C10 mature effectors of monocytes. At first, cluster C7 captures the initial primed state in the blood, characterized by the up-regulation of early damage-associated molecular patterns (DAMPs) like S100A12, alongside cytoprotective protease inhibitors (CSTA and SERPINB1) that prevent premature degranulation [67,68]. After that, C8 reflects a transient margination and rolling phase, distinctly marked by the expression of the SELL and the chemotactic sensor FPR1 [69]. Following the rolling phase, C9 marks the critical transition where the monocyte slows down and breaks the endothelial barrier. To achieve this adhesion, the cells up-regulate the essential integrin named ITGAM gene. After that, the monocyte must physically remodel its structure to break through the vessel wall and this process is supported by TPM4 and GNAQ [70]. Simultaneously, while crossing, C9 monocytes act as local coordinators by expressing the chemokine CXCL8 to recruit subsequent neutrophils [71]. Finally, upon entering the inflamed tissue, the monocyte cascade culminates in cluster C10, representing the fully mature effector and antigen-presenting state. This stage is characterized by the activation of the inflammasome CASP1 and OSM, which actively regulate local tissue inflammation [72]. Moreover, the metabolic reprogramming necessary fulfills energy demands, which is observed in SLC2A3, and the up-regulation of molecules essential for T cell interaction (CD86 and HLA-DRB1) [73]. This path, which shows activations of monocytes in the inflammatory process, proves the biological relevance of discovering subclusters by GMM with K-M.

### Simulated scRNA-Seq data

**Fig. 6.**
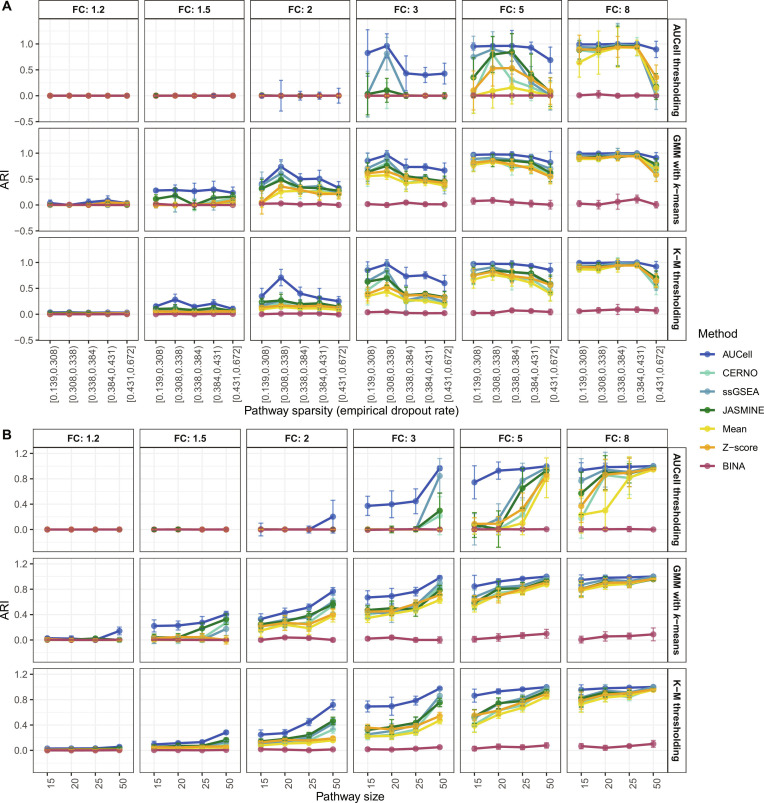
Investigation of pathway size and sparsity for tested ssEA methods and ATDs on simulated datasets. (A) Results for pathway sparsity effect. (B) Effect of pathway size. On both panels, the fold change (FC; sample separation) is distinguished. Each point represents the median value, and the error bar shows its 95% confidence interval.

To validate observed findings in the control experiment, the generated scRNA-Seq datasets of known various pathway sizes, as well as different FCs between groups, which impact pathway sparsity, were used. At first, the log normalization was considered, since this type of normalization was applied for all real biological data. The results of the experiment are presented in Fig. 6. As can be observed for FC = 1.2, regardless of ssEA and ATD, there are no differences between results. With the increasing separability between groups (larger cell activity; increasing FC), GMM with *k*-means and simple *k*-means work more effectively compared to gold standard AUCell thresholding. This confirms the previous observation on real data. Yet, the simulated experiment was performed on a much larger dataset collection (4 different pathway sizes, 6 different FCs, 5 replicates, in total, 120 datasets). Next, it can be observed that with increasing pathway sparsity, the ssEA methods (regardless of ATDs) decrease their performance. The opposite can be observed for pathway size, where ssEA methods work better for larger pathways. This is the effect of data compression and better noise reduction when more genes are considered. This experiment confirms that GMM with *k*-means and simple *k*-means are better ATD techniques compared to AUCell thresholding, especially with the rank-based ssEA. Surprisingly, rank-based methods (especially AUCell) perform better than Mean and Z-score regardless of ATDs. Again, the effect of ssEA method in the detection of relatively active cells/samples is lower than the usage of a correct ATD technique. During the analysis of simulated data, the BINA method performed extremely poorly, in contrast to JASMINE. While JASMINE shows low effectiveness on real datasets, the simulations suggest the opposite. Consequently, based on the results of this study, the use of both JASMINE and BINA should be approached with caution. Nevertheless, despite the poor performance of new ssEA BINA on simulated data, its results are included to demonstrate that a single dropout-based measure is insufficient for pathway enrichment analysis in scRNA-Seq data. Finally, the effect of normalization was investigated, and previously used log normalization was compared to SCTransform (Fig. S10). First, it can be observed that results for AUCell thresholding are characterized by much larger variance between the normalization methods compared to the proposed ATDs. This indicates better robustness to data pre-processing of the proposed ATD methods. Next, it can be observed that rank-based ssEA methods are rather robust to the data transformation, and sometimes, they work better on simple log normalization, especially for proposed ATDs and low matrix sparsity. On the contrary, Mean and Z-score perform better after SCTransform, and the trend to pathway matrix sparsity is observed. Finally, the pathway size does not influence ATD performance. Yet, the parametric ssEA methods, again, work better for SCTransform with the increasing pathway size. Nevertheless, despite the superior results observed for Mean and Z-score under SCTransform normalization, the conclusions drawn from the biological data analysis remain valid; under log normalization, the only ssEA algorithms that yielded statistically significant differences when compared to one another were JASMINE and BINA, which are characterized by poor outcomes.

### Computational time

The first computational time scenario compared the runtime for analyzing all pathways at once (red dashed line in Fig. S11A) versus a single pathway (boxplots in Fig. S11A). ssGSEA was slowest when pathways were run separately due to repeated ranking, but faster when run jointly, while JASMINE showed the opposite. Nevertheless, the process of calculating PAS is relatively fast. Next, the relation between pathway size and time was investigated (Fig. S11B). Only Z-score and ssGSEA showed no pathway size versus time relationship; for other algorithms, larger pathways required longer runtimes, as expected from their complexity. Finally, the computational load of the ATDs was evaluated (Fig. S12). As expected, GMM thresholding exhibited the longest execution time due to the complexity of mixture model decomposition. While GMM represents the most computationally demanding approach in our comparison, its mean processing time of 21.3 s remains well within practical limits for HT analysis. In contrast, AUCell thresholding was notably faster, with an average duration of 2 s, while the most efficient method, simple *k*-means, achieved best performance at 0.3 s. The variations in execution time for individual ATD methods across different ssEA are negligible.

### Other HT data application

Finally, we applied ATD to other HT transcriptomic methods using HER2^+^ breast cancer subtype signature and microarray, bulk RNA-Seq, and scRNA-Seq data. All ssEAs (except BINA, which relies on zero counts and is incompatible with microarray data) and all ATD methods were re-evaluated using ARI and FDR (Fig. S13). As can be observed, the AUCell thresholding, regardless of HT technique and ssEA, gives the worst results. This may be the effect of a much smaller sample size. For the bulk RNA-Seq, it can be noticed that GMM with K-M gives the highest median ARI and reasonable FDR compared to other ATD methods. Yet, for scRNA-Seq, which is twice smaller than the bulk experiment, the K-M solution outperforms others. Finally, for the microarray dataset, which was the smallest, the K-M and GMM solutions performed similarly. The ARI values were lower than in previous sections, yet consistent with reduced detection power (Fig. S13A, gray dashed line). Nonetheless, the results remain promising for other HT experiments.

Lastly, the scRNA-Seq experiment of large resolution (over 80,000 cells) was used for testing. The best possible ARI in detecting GAMs was calculated, and the results are presented in Fig. S14. For Fig. S14A and B, the best ARI is usually obtained by the K-M method. Yet, due to the large dataset, optimal K was not searched, and only 2 clusters were considered. Interestingly, rank-based ssEA methods have a better ability to reflect activation of the pathway than the rest. Again, JASMINE enrichment performs the worst. The proposed BINA, which previously also had poor outcomes with a combination of K-M, shows good results. Lastly, GMM with K-M does not show relatively good performance, but combined with CERNO, it shows the ARI on the same level as the best possible to obtain.

## Discussion

We systematically evaluated relative pathway ATD techniques and present a framework called FUNCellA. Although this type of pathway-based clustering was previously proposed in the AUCell [11], it has not been systematically evaluated. Furthermore, the previous solution is complex, GMM decomposition is limited, and the output only enables binary classification of active or inactive cells, and, as shown here, is not effective. We proposed alternative solutions based on *k*-means clustering and GMM combined with *k*-means, which are fully unsupervised in searching for the number of cell clusters and not limited to binary decisions. In the presented research, the AUCell thresholding, together with proposed ATDs, was applied to 7 different ssEA methods, and guidance on their optimal combinations is presented. The benchmarking was conducted on real scRNA-Seq data, simulated data, and data from other transcriptomic HT data. All presented methods are implemented and widely available in 2 programming languages, as well as a web application. Finally, to the best of the authors’ knowledge, the proposed benchmarking approach for assessing the relative activity of a pathway in samples is novel. Furthermore, the 2 proposed ATDs, i.e., *k*-means and GMM with *k*-means, represent a novel application of these algorithms to the specific problem of detecting relative pathway activation within biological samples.

The pathway-based enrichment analysis is a key approach in bioinformatic analysis for uncovering biological and molecular mechanisms. With the development of scRNA-Seq, an increasing number of single-cell investigations have adopted PAS analysis to explore cellular heterogeneity [74,75]. Presented solutions concentrate only on ssEA, for which a higher PAS value indicates higher pathway activity in a particular sample. For this purpose, a group of strong marker pathways was benchmarked on a well-described PBMC dataset and validated on other scRNA-Seq experiments. At first, we noticed that different ATD methods work differently regarding the ssEA. Thus, we categorized them into 3 groups: (a) rank-based, i.e., AUCell, CERNO, ssGSEA, and JASMINE; (b) parametric-based, i.e., Mean and Z-score; and (c) proportion-based, i.e., BINA. Furthermore, we demonstrated that in the majority of the benchmarking process, AUCell thresholding does not outperform the proposed ATDs, as is clearly visible in Figs. 2, 3, and 6 or Fig. S3. This observation is independent of the ssEA. When other HT transcriptomic experiments were checked, AUCell thresholding had ARI below 0.1 and was significantly worse than the proposed solutions (Fig. S13). Yet, AUCell thresholding began to yield good results when applied to a large scRNA-Seq experiment, where results were very close to the best possible to obtain. Yet, K-M ATD also shows similar performance. Through the proposed solutions for searching relative pathway activation, we can observe that GMM Top1 detects very strict activation and should not be used unless the study design does not assume small activations or the detection of rare activated subpopulations. Next, we observed that for small scRNA-Seq studies (from ~3,000 to ~14,000 cells tested), the GMM with K-M is effective for rank-based ssEA, while K-M ATD is suitable for parametric-based approaches. Further, the simulation data confirm the effectiveness of the proposed ATDs. What is more, this experiment was enriched by introducing another data normalization technique. The process of data normalization can highly impact ssEA [21]. Nevertheless, other authors (Andreatta and Carmona [31]) have shown that rank-based enrichments are robust to applied normalization and data composition. Our results demonstrate that indeed rank-based ssEAs are more robust to data transformation, while Mean and Z-score may achieve better results on SCTransform. In addition, it was shown that proposed ATDs give lower variance of results between different normalizations, indicating that they are more resilient to pre-processing. Next, in comparison with ssEA combined with their optimal ATDs, no statistical significance was observed between rank and parametric-based approaches (Fig. 4 and Fig. S8). However, a noteworthy limitation of the presented benchmark is that it inherently assumes uniform pathway activity across broadly annotated cell types. Nonetheless, because single-cell populations are heterogeneous, algorithms that correctly identify subpopulation-specific pathway inactivity (e.g., within diverse T cell subsets or B cells) may be inadvertently penalized. Thus, only the simulated data experiment allows for unbiased assessment of ssEA and ATD performance.

When a large study from scRNA-Seq was analyzed (GAM cell detection), the K-M outperformed other methods regardless of ssEA. However, due to computational intensity of calculating the Silhouette index, *K* was fixed and set to a value of 2. In contrast for GMM with K-M, the full search of clusters was performed using AIC. This highlights a key limitation of the *k*-means ATD approach, which is the computational cost of traditional cluster selection indices in large-scale data. This constraint is less pronounced for AUCell thresholding or the GMM–K-M approach, as in the latter, *k*-means is only applied within the GMM parameter space.

Further, compared to K-M and AUCell thresholding, the GMM with K-M can show mid activations as full decomposition of the signal is available (Fig. 5). Moreover, the detected clusters are not random and carry the step-by-step progress of inflammatory response in monocyte cells. The presented benchmark suggests how to combine ATDs with ssEA. The process of sample grouping via pathway activation determination can be applied not only to cell grouping but also to all omic data, e.g., it can detect heterogeneous pathway activity that may lead to different drug responses.

One potential concern of application of FUNCellA is a multi-sample scRNA-Seq experiment, where the confounding effect of sample-specific variation is observed. It is important to emphasize that a fundamental condition for the effective use of FUNCellA is a rigorous data pre-processing pipeline that address technical batch effects at the gene expression level before scoring pathway activity. Yet, the transition from sparse, noisy gene counts to PASs especially via the rank-based method, such as ssGSEA, CERNO, or AUCell, acts as a partially natural denoising and stabilizing step. In FUNCellA, we intentionally employ a global thresholding approach across the entire dataset. While this could theoretically be influenced by interpatient variability, applying a unified global distribution for clustering ensures that identified cellular states are comparable across all samples.

Finally, computational time is a critical point in analyzing the large molecular biology data. We show that the execution time of the ssEA method is similar to tested algorithms and acceptable for this problem as it takes around 10 s or less. The most expensive is ssGSEA (30 s by average), but when it is run for multiple pathways at once, the time significantly decreases (20 s). In terms of ATDs, the *k*-means approach is the fastest solution with time less than 1 s per pathway. Next, the AUCell thresholding with average 2 s and GMM with *k*-means can be distinguished (21.3 s). The time load for GMM with *k*-means is the longest due to the GMM decomposition by histogram partition.

To summarize, we introduced a freely available tool called FUNCellA with 2 new relative pathway ATD techniques (based on *k*-means and GMM connected with *k*-means). Both *k*-means and GMM are commonly known unsupervised methods but their application in presented solution is novel, especially the combination of GMM and *k*-means. This automatic unsupervised solution helps users to determine the threshold of relative pathway activation, which in UCell package tutorials is set manually. Furthermore, the only one unsupervised gold standard AUCell thresholding performs significantly worse compared to proposed solutions. Yet, the FUNCellA package allows to use all the tested ATDs together with all tested ssEA as a wrapper implementation. In addition, we proposed a novel benchmarking method to assess the performance of relative pathway activation. Among the tested ATD methods, the simple *k*-means thresholding can be highly effective in many cases. When rank-based enrichment algorithms are used, the GMM with K-M is potentially more informative, as it divides the signal into sub-activations, which may provide more detailed insights into biological processes.

## Data Availability

The FUNCellA package is freely available as an open-source R package at https://github.com/ZAEDPolSl/FUNCellA, and a Python package at https://github.com/ZAEDPolSl/pyFUNCellA. The web application is accessible at https://dssoftware.aei.polsl.pl/FUNCellA/. The analyses are based on publicly available datasets: E-MTAB-9580, E-MTAB-9221, GSE132044, GSE159977, E-GEOD-70951, GSE182109, GSE75688, and TCGA-BRCA cohort available via the GDC portal. Simulated data are available upon request.
