# Supplementary material for: FUNCellA: A Tool for Single-Sample Enrichment Analysis and Relative Pathway Activity Estimation in Single-Cell RNA Sequencing Data
Source: Comput Struct Biotechnol J. 2026 Apr 21;35(1):0053. doi: 10.34133/csbj.0053 (PMC13096679; doi:10.34133/csbj.0053)
Supplement: Supplementary 1 — Figs. S1 to S14 Tables S1 to S8 [file csbj.0053.f1.docx]

**Supplementary Materials**

**FUNCellA: a tool for single-sample enrichment analysis and relative pathway activity estimation in single-cell RNA sequencing data**

Joanna Zyla^1,*^, Anna Mrukwa^1,+^, Aleksandra G. Bilska^2,3,+^, Kamila Szumala^4^, Joanna Polanska^1^ and Michal Marczyk^1,5^

^1^ Department of Data Science and Engineering, Silesian University of Technology, Gliwice, Poland, ^2^ Doctoral School of Molecular Medicine, Medical University of Lodz, Lodz, Poland, ^3^ Computational Biology Lab, Institute of Computer Science of the Polish Academy of Sciences, Warsaw, Poland, ^4^ Department of Systems Biology and Engineering, Silesian University of Technology, Gliwice, Poland, ^5^ Breast Medical Oncology, Yale Cancer Center, Yale School of Medicine, New Haven, CT, USA,


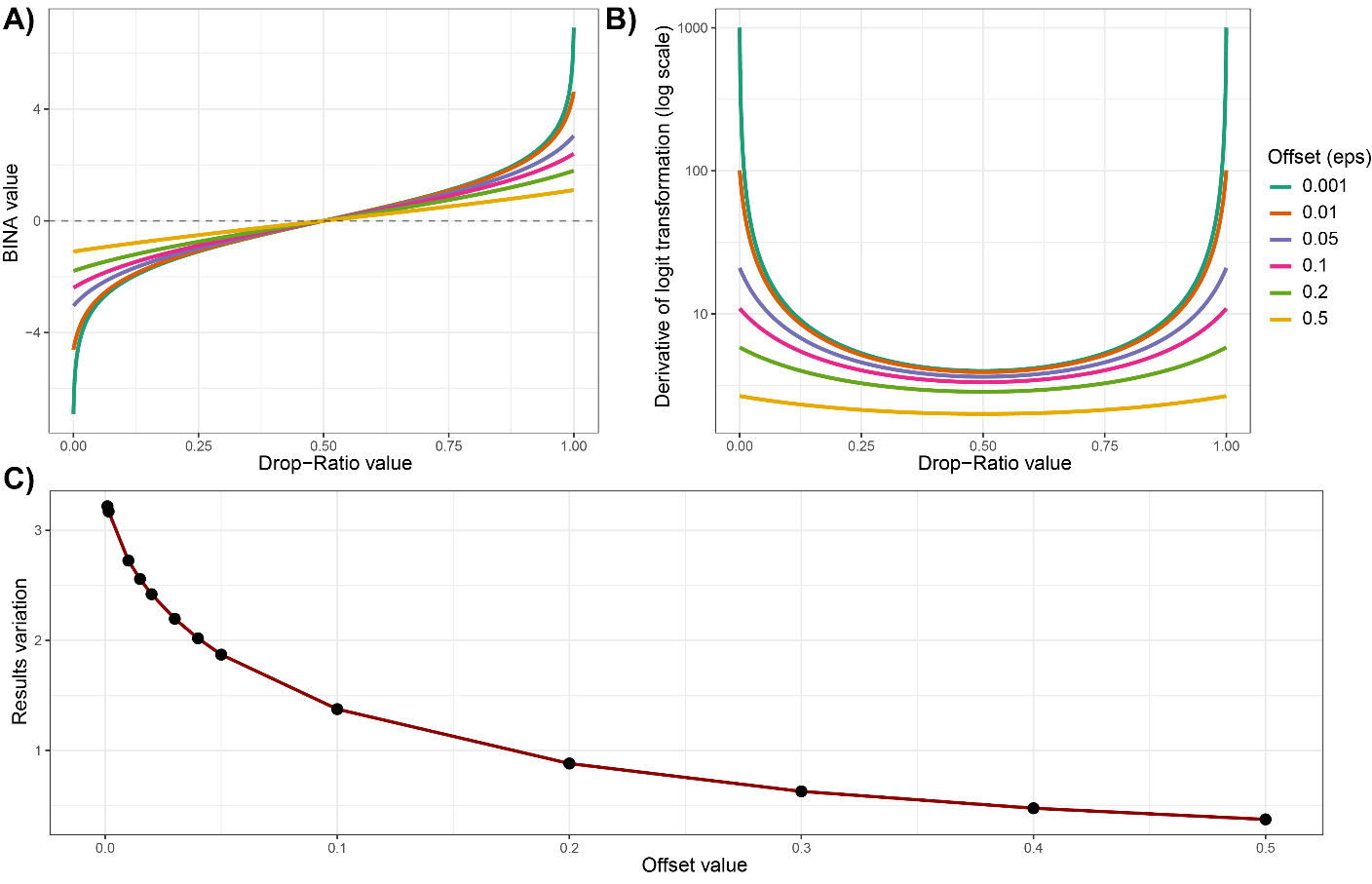


**Supplementary Figure 1. Sensitivity analysis of offset value in BINA.** Panel A and B show relation between drop-ratio versus logit transformation and its derivative, respectively. Panel C shows impact of offset into the results variation.


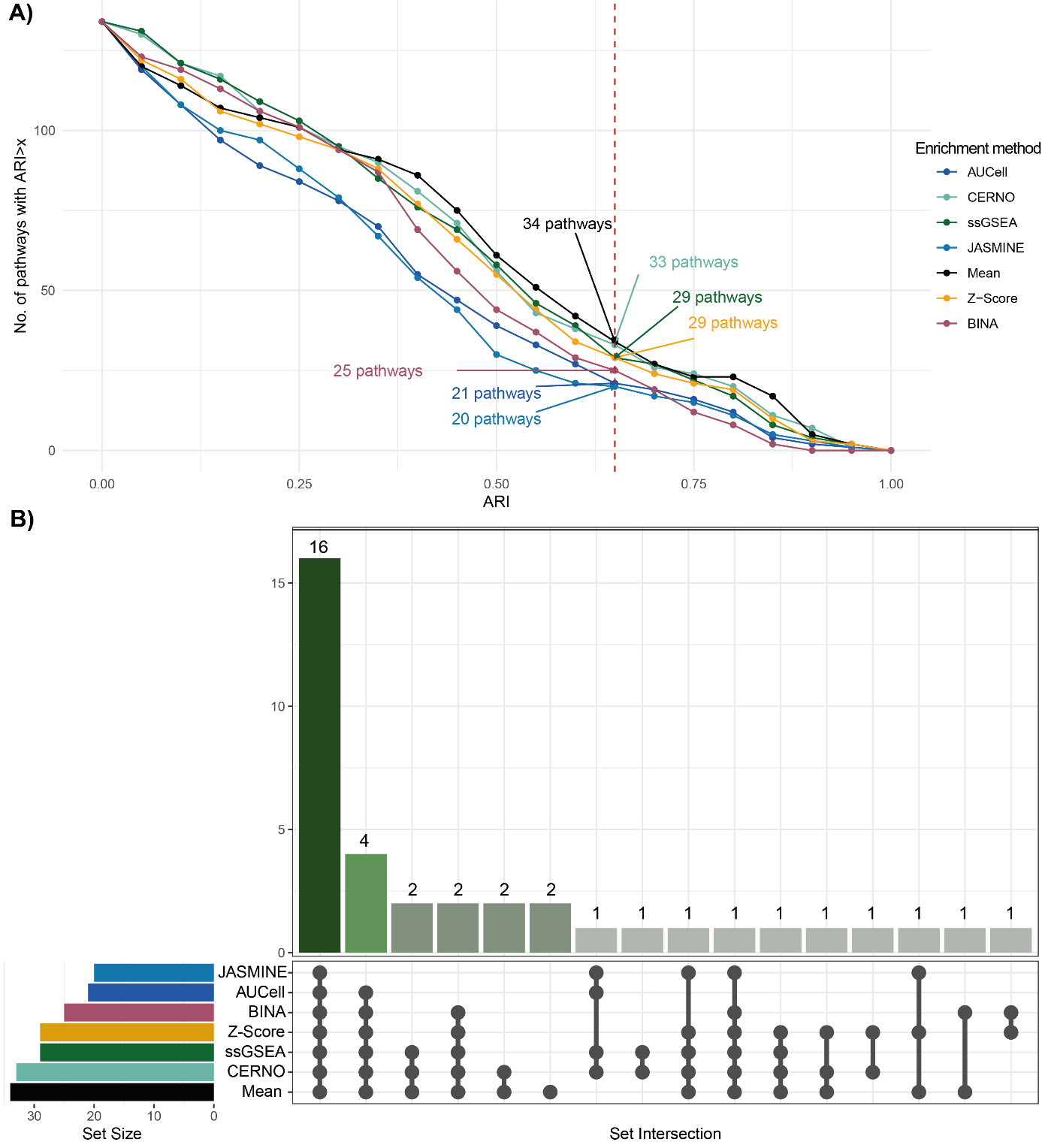


**Supplementary Figure 2. Summary of pathways selection used in study.** Panel A shows drop of pathway number under various cut of ARI level for optimal threshold. Panel B shows intersection of pathways between enrichment methods for selected moderate recovery at level 0.65 cutoff.


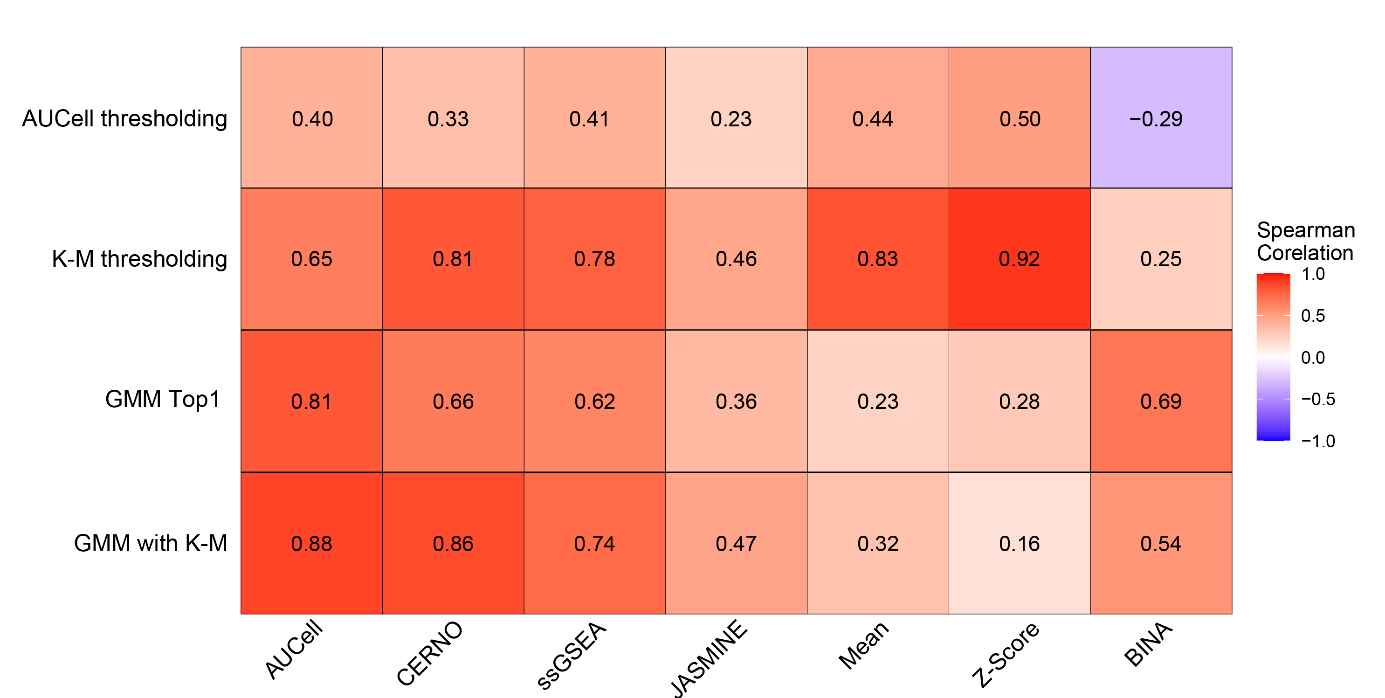


**Supplementary Figure 3. Correlation between the best ARI obtained from perfect fit probing and those given by ATD within each enrichment method separately.**

**
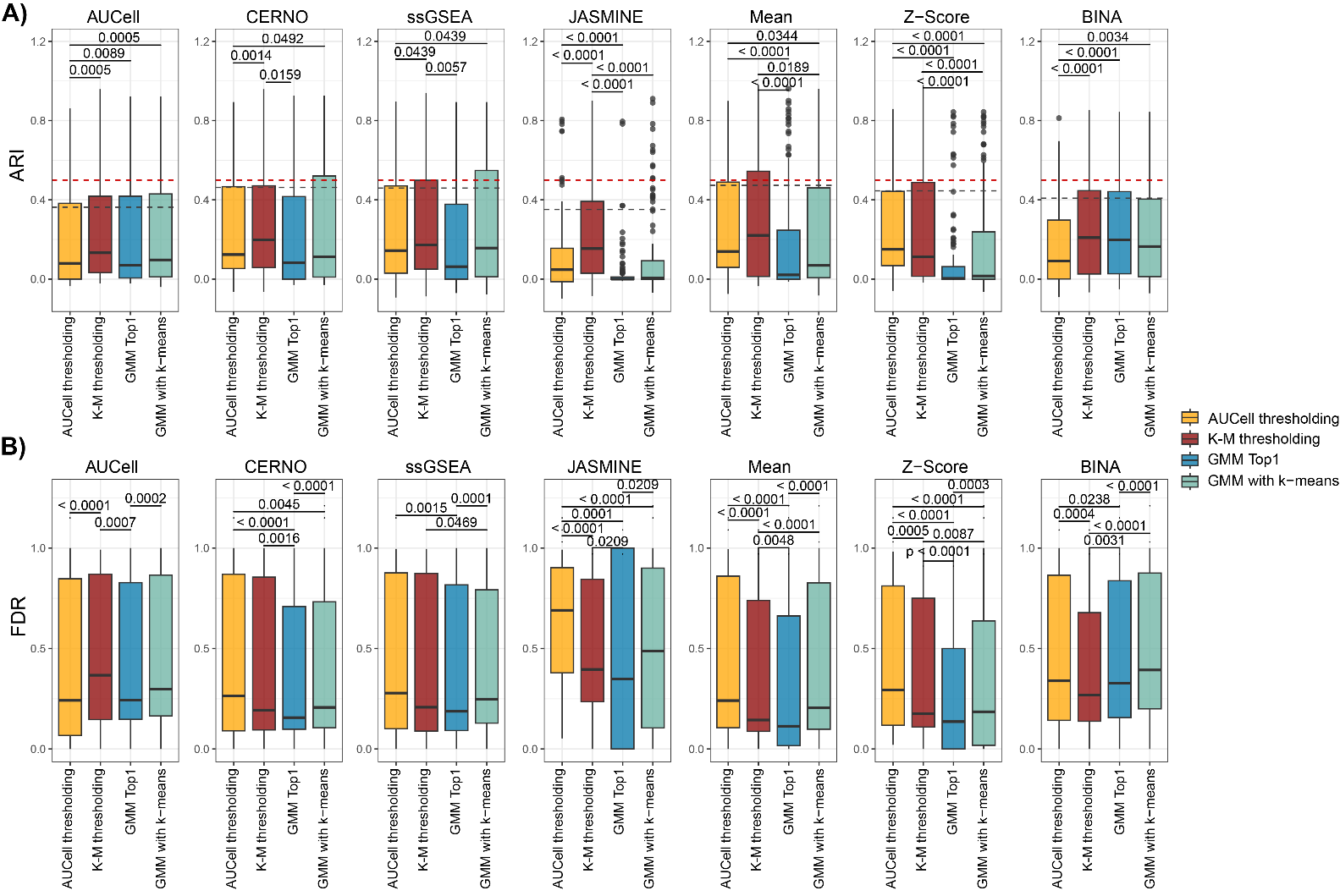
**

**Supplementary Figure 4. Results of activation threshold determination methods across tested ssEA for PBMC dataset without selection of ARI level.** On the Y axis on panel A the adjusted rand index metric result is presented while on panel B for false discovery rate. For both panels, X axis corresponds to ATD method which is encoded in colour as well. On panel A red dashed line represents ARI at 0.65 (which corresponds to the moderate recovery), while the grey dashed line represents the median value of ARI for the best possible threshold. The presented p-values were obtained by pairwise Wilcoxon signed-rank test comparison for paired samples, and only statistically significant outcomes are marked. For each ssEA 134 pathways were investigated.


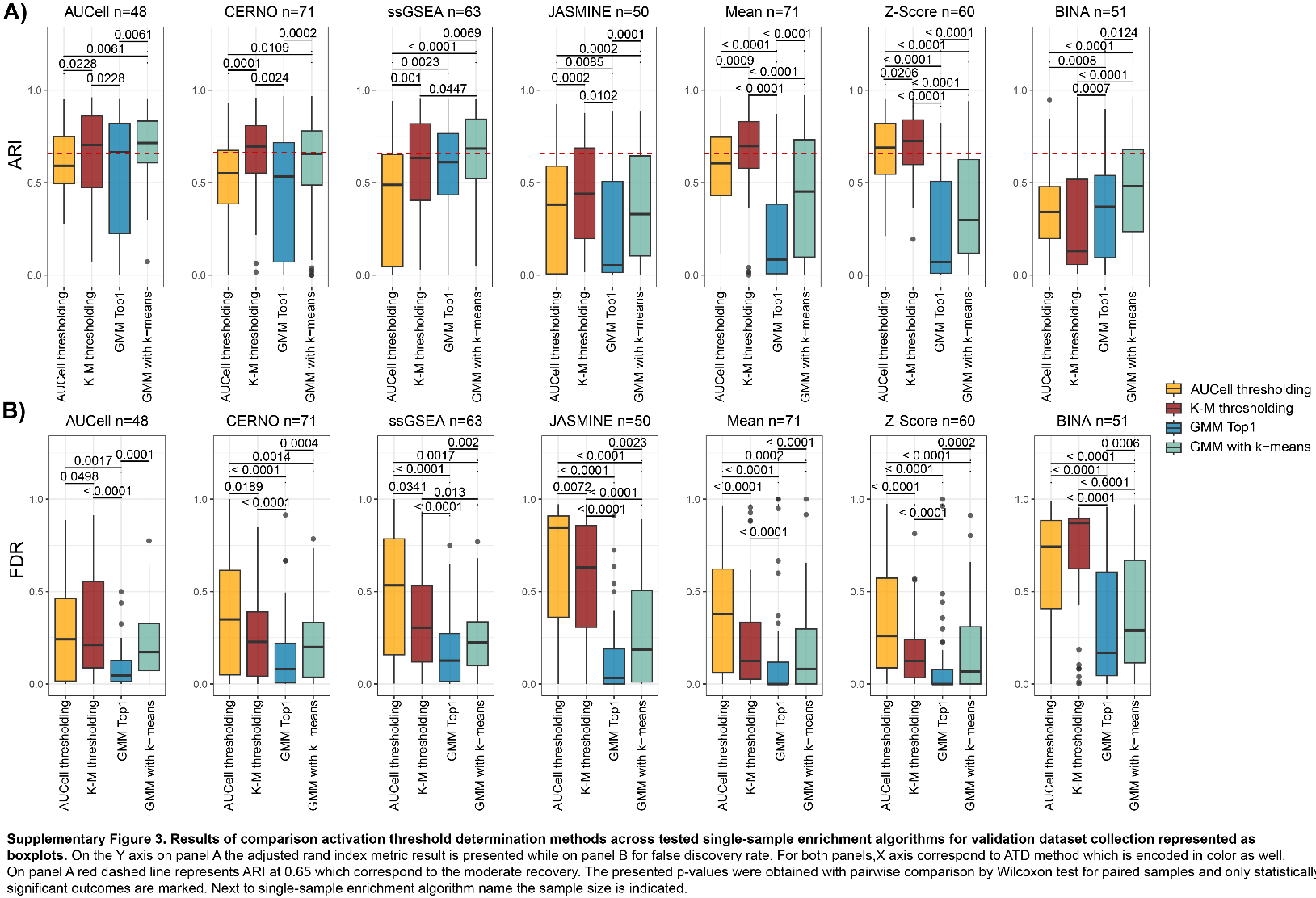


**Supplementary Figure 5. Results of activation threshold determination methods across tested ssEA for validation dataset collection.** On the Y axis on panel A the adjusted rand index metric result is presented while on panel B - false discovery rate. For both panels, X axis corresponds to ATD method which is encoded in colour as well. On panel A red dashed line represents ARI at 0.65 which corresponds to the moderate recovery. The presented p-values were obtained by pairwise Wilcoxon signed-rank test comparison for paired samples, and only statistically significant outcomes are marked. Next to ssEA name, the sample size is indicated.

**
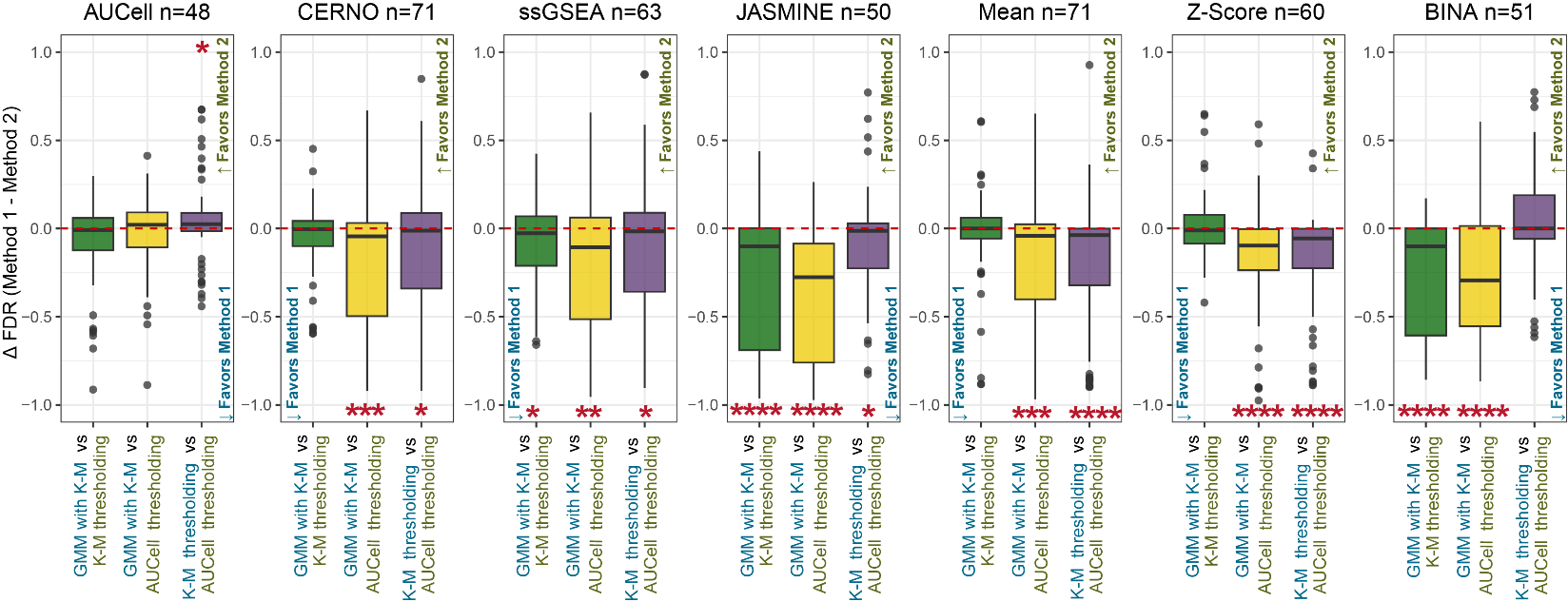
**

**Supplementary Figure 6. Difference of FDR metric between activation threshold determination methods across tested single-sample enrichment algorithms for the validation set.** The Y-axis represents the difference calculated as Method 1 minus Method 2, which corresponds to the first and second algorithms listed in each comparison pair on the X-axis. As FDR represents an error rate, negative values indicate better performance of Method 1, whereas positive values favor Method 2. The significance is marked as follows: * p<0.05, ** p<0.005, *** p<0.001,**** p<0.0001.

**
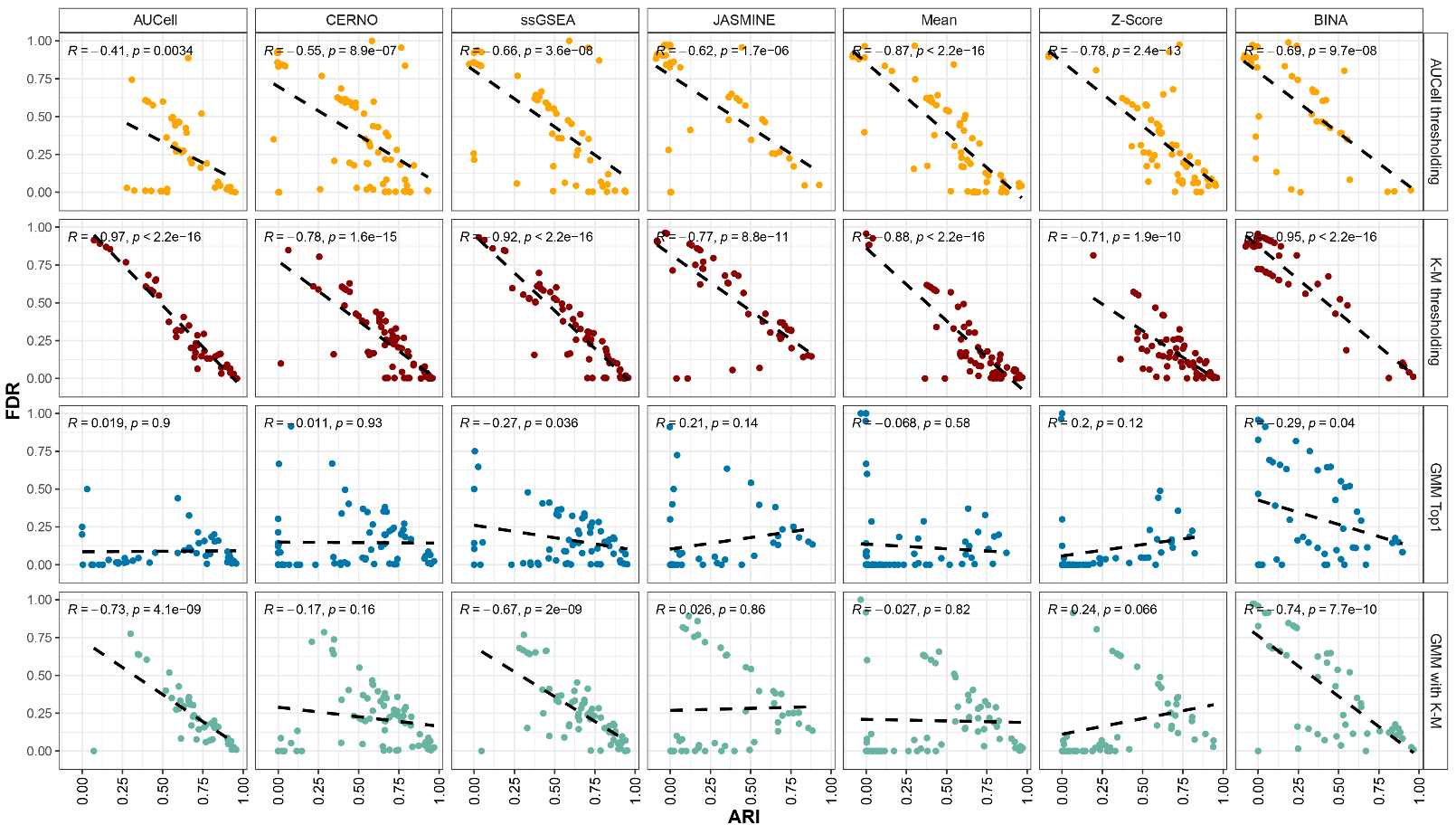
**

**Supplementary Figure 7. Relation between ARI and FDR for validation experiment.** The marked R stands for Spearman correlation coefficient, while p shows the p-value for the correlation.

**
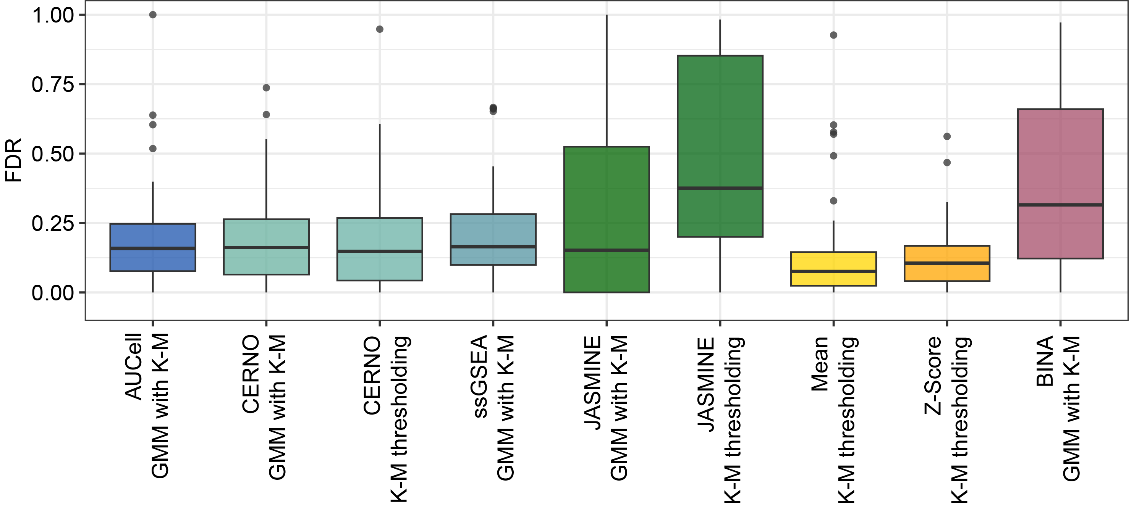
**

**Supplementary Figure 8. False Discovery Rate of tested single-sample enrichment algorithms with their best activation threshold determination technique for all common pathways in immunological datasets.**


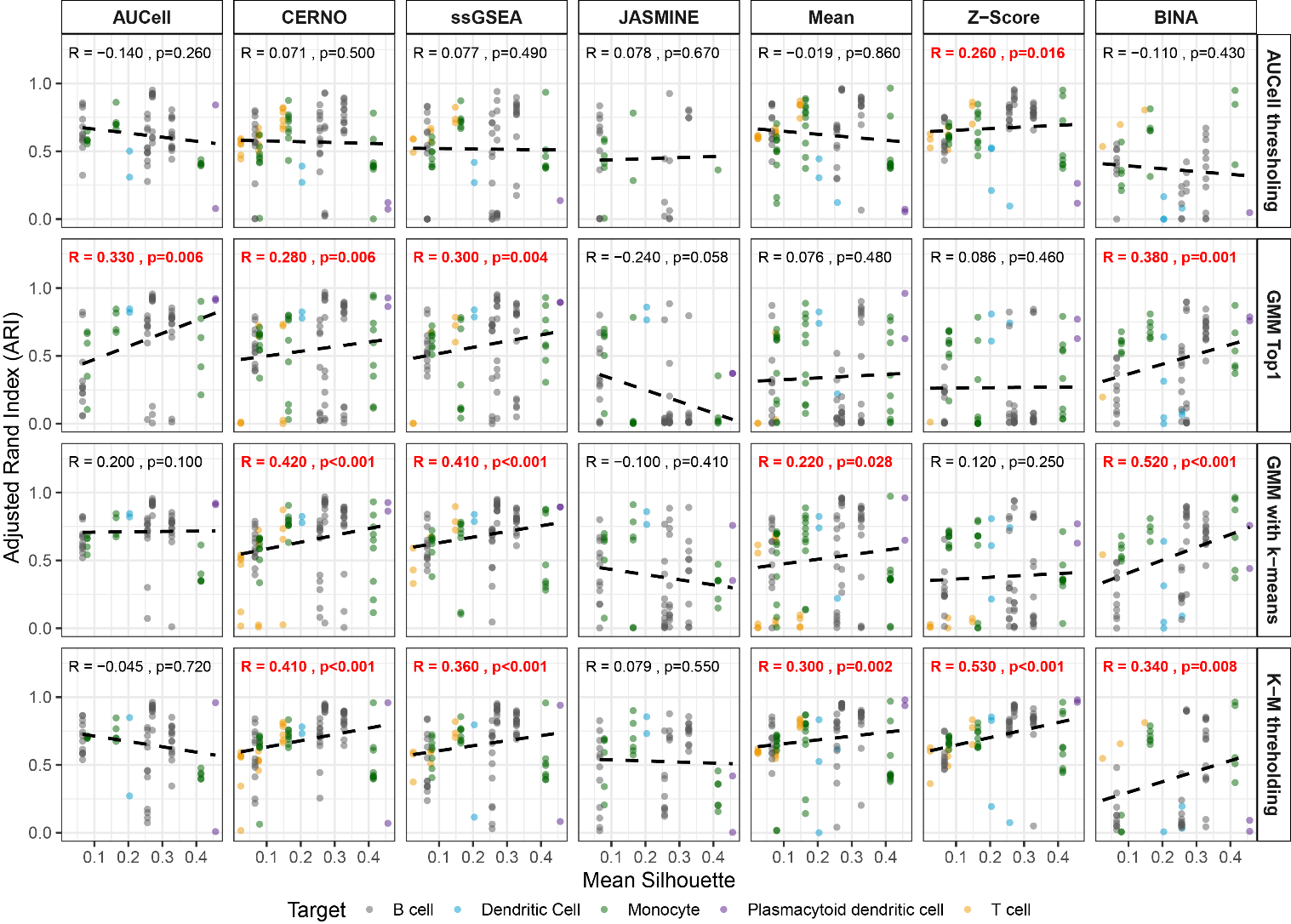


**Supplementary Figure 9. Relation between ARI and average Silhouette width of cells for all datasets.** The marked R stands for Spearman correlation coefficient, while p shows the p-value for the correlation. The red color of the correlation and p-value indicates statistical significance.

**
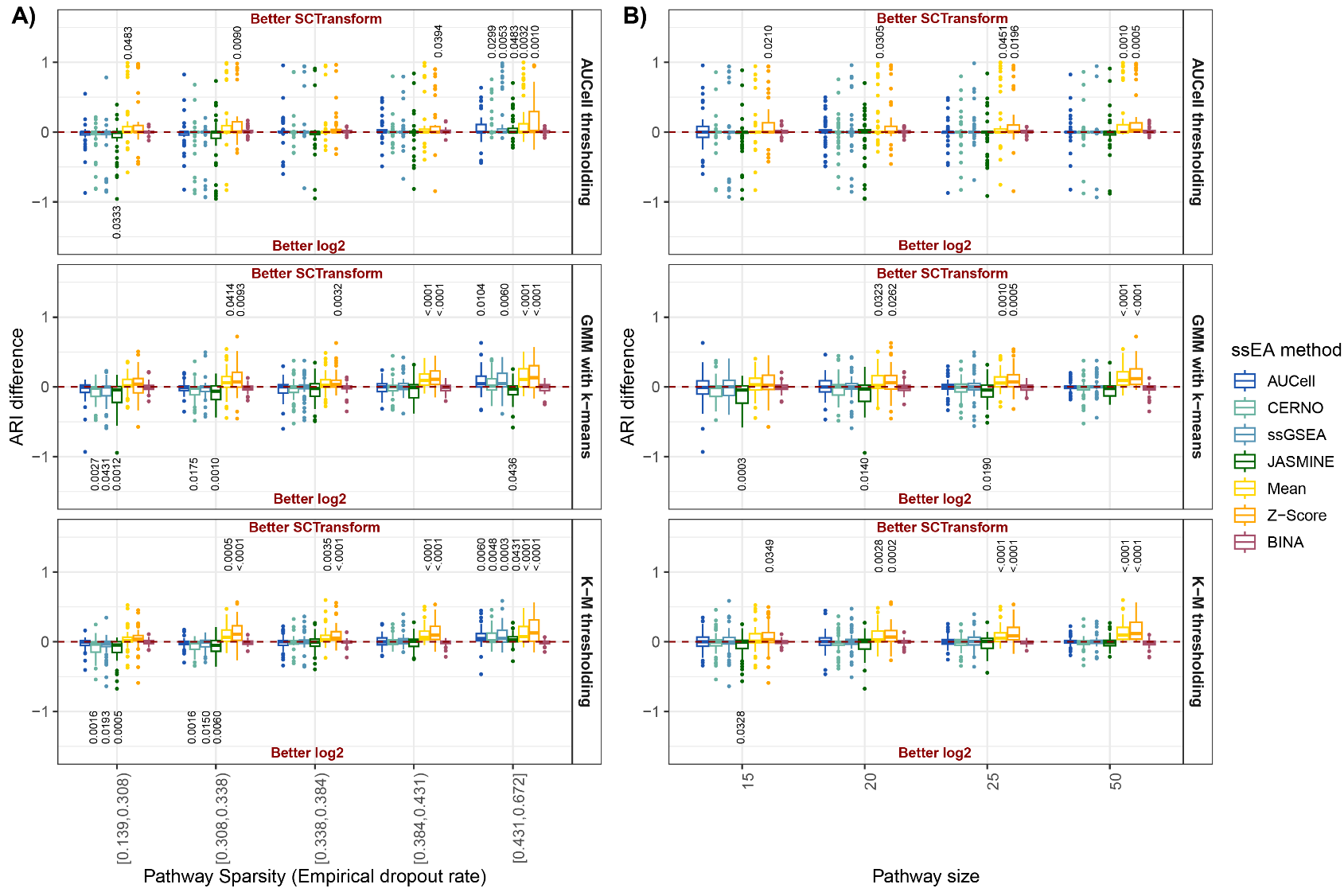
**

**Supplementary Figure 10. Impact of normalization on tested ATD and ssEA performance.** Comparison of SCTransform vs. log-normalization across pathway sparsity (A) and pathway size (B). The Y-axis represents the ARI difference (values > 0 favor SCTransform). Results are shown for three thresholding methods and seven enrichment algorithms. Statistical significance was calculated using a two-sided Wilcoxon signed-rank test against a median of zero. P-values are indicated only for significant comparisons.

**
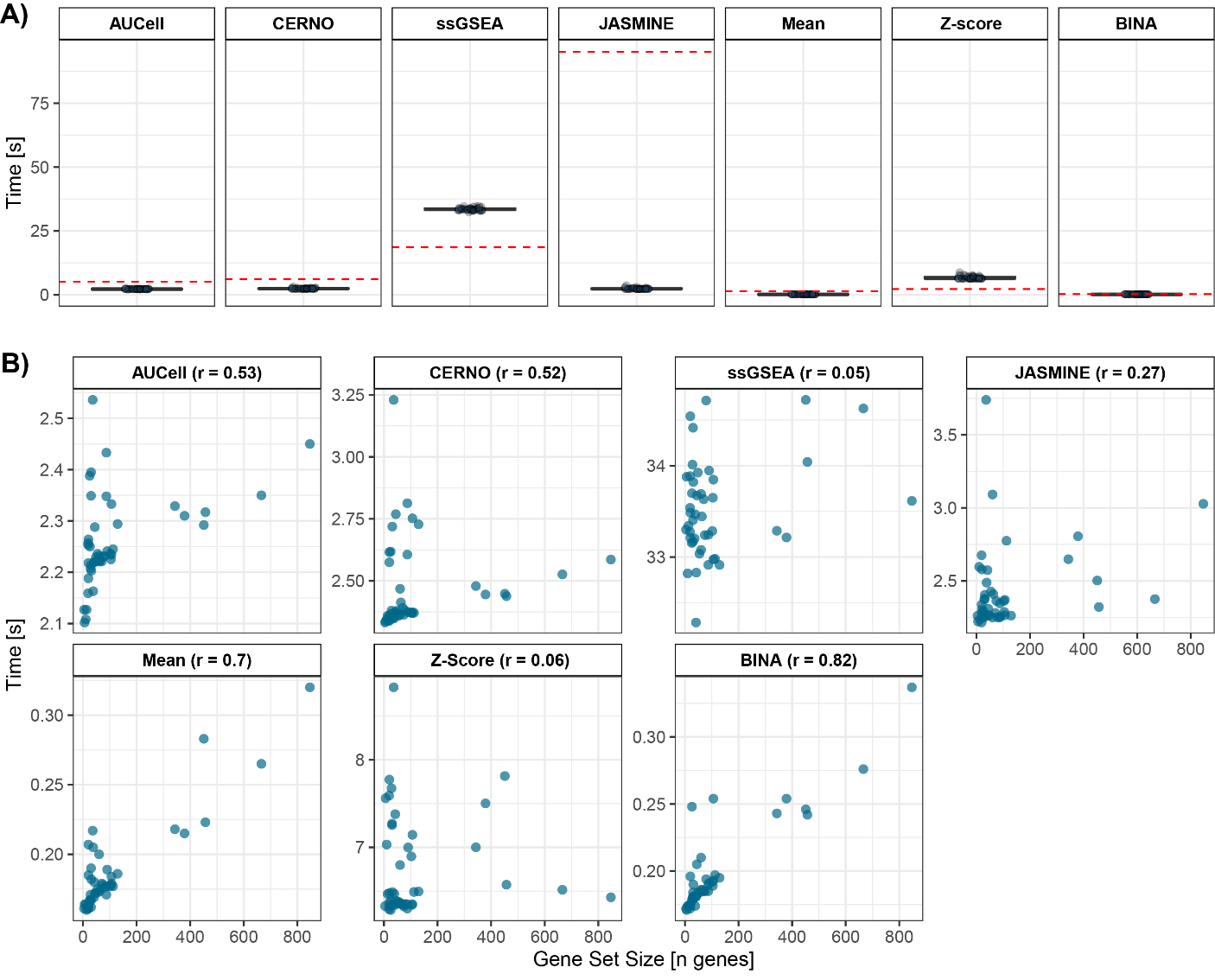
**

**Supplementary Figure 11. Results of computational time investigation.** Panel A shows the computational time (in seconds) for each tested enrichment method. The boxplots represent the distribution of computational time for single pathway analysis, while the red dashed line indicates the computational time for entire collection analysis. Panel B illustrates the relationship between computational time and pathway size. The Spearman correlation coefficient, reported in the panel heading, quantifies this relationship.


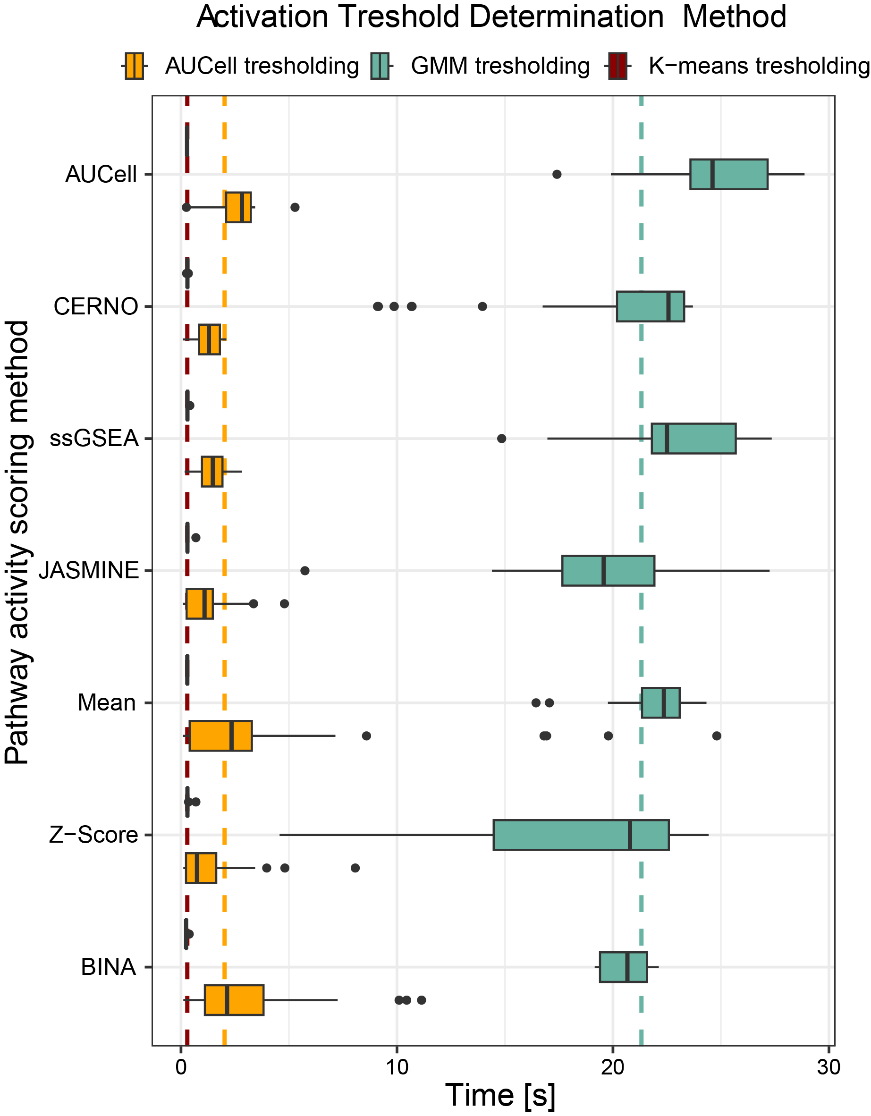


**Supplementary Figure 12. Results of computational time investigation for ATD methods across different ssEA algorithms and 45 various pathways.** The vertical lines represent average time regardless of ssEA algorithm for each ATD method.


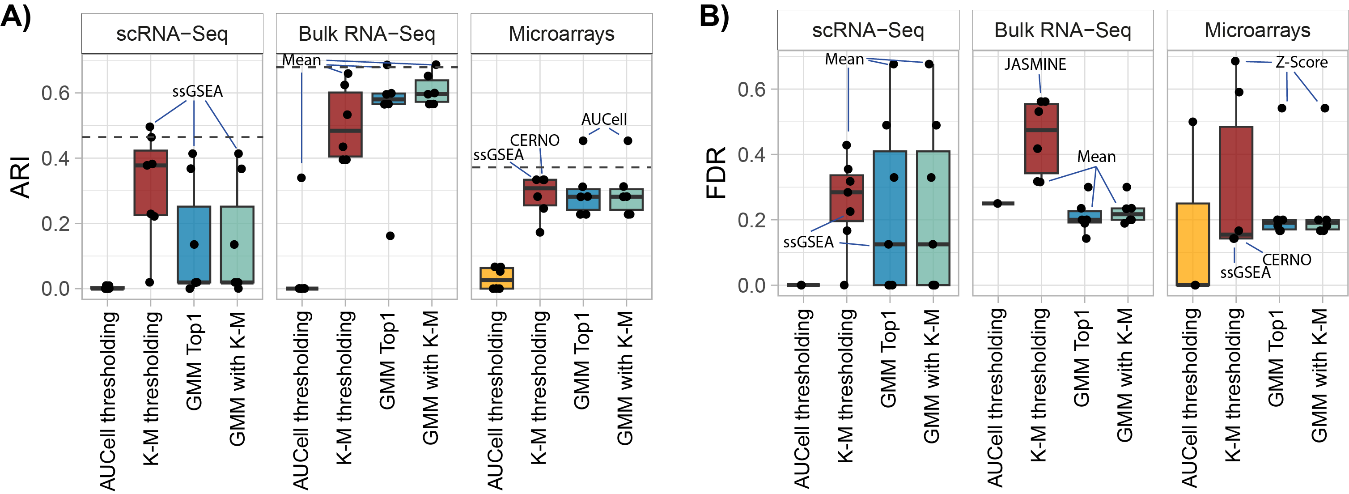


**Supplementary Figure 13. Performance of activation thresholding determination methods on different HT techniques of small sample size.** On the Y-axis of panel A, the adjusted Rand index is presented, while on panel B, the false discovery rate. For both panels, the X-axis corresponds to ATD method, which is color-coded. The grey dashed line on Panel A represents the median of the best possible outcomes.


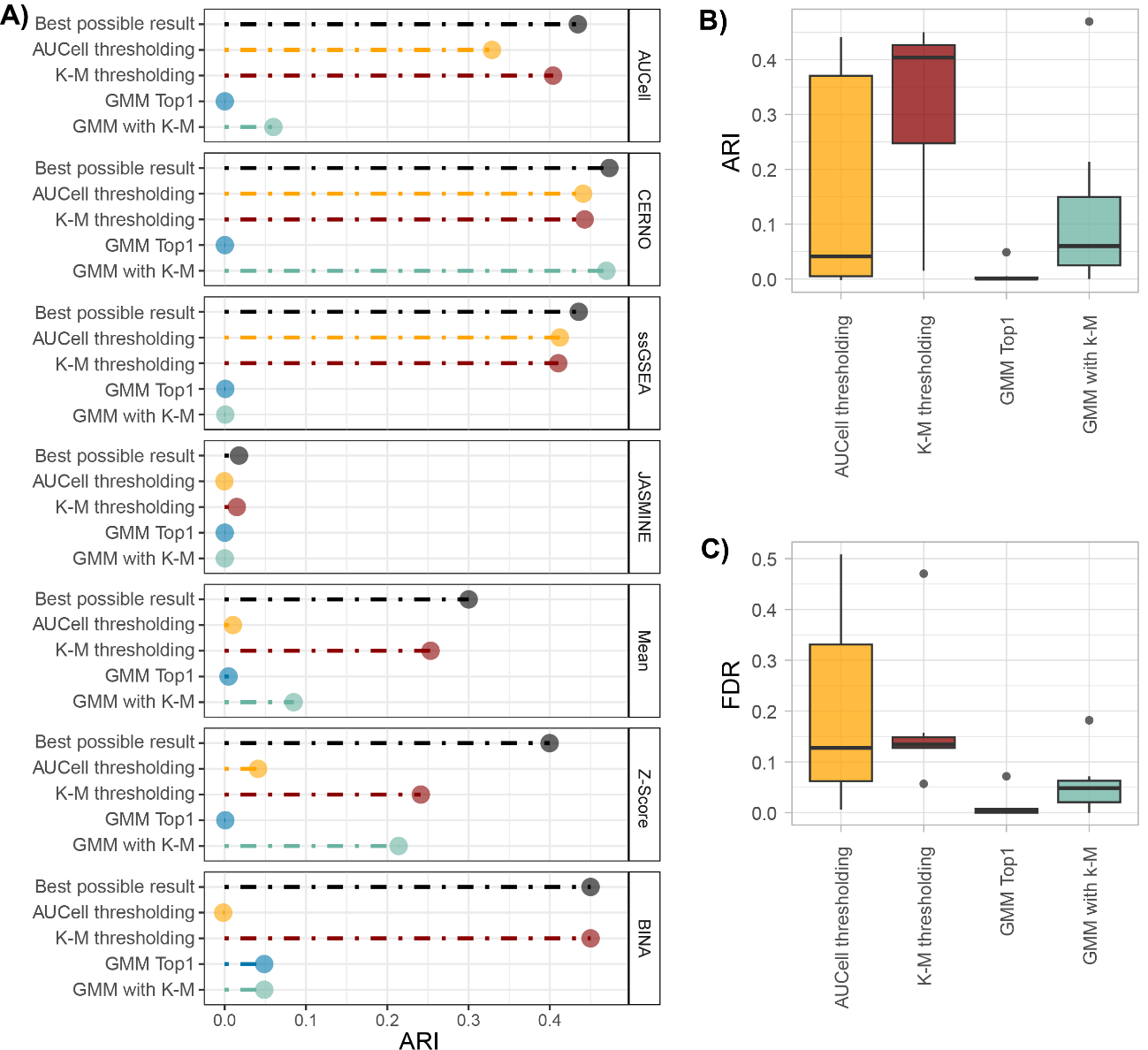


**Supplementary Figure 14 Results of evaluation metrics for Abdelfath dataset.** Panel A shows ARI for each tested single-sample algorithm and ATD method including the best possible result (black colour). Panel B and C shows ARI and FDR respectively regardless of single-sample algorithm.

**Supplementary Table 1. Detail summary of used ssEA methods.**

| **Method** | **scRNA-Seq dedicated** | **Previously published** | **PAS calculation approach** | **Tested run** |
| --- | --- | --- | --- | --- |
| AUCell | ✓ | ✓ | AUC for each sample for 5% top ranked genes. | Function *AUCell_buildRankings* from AUCell package with default parameters and suggested by authors aucMax:   - aucMaxRank=0.05*nrow(X) |
| CERNO | X / ✓ Original CERNO was not dedicated to scRNA-Seq but the same method UCell was published later and validated as proper for scRNA-Seq | ✓ | AUC score based on the Mann-Whitney approach on ranked genes. | FUNCellA implementation; function gene2path.  No parameters. |
| ssGSEA | X | ✓ | Enrichment Score based on gene rankings for each sample. Biggest absolute ES as final score for the pathway and sample. | Function *ssgseaParam* from GSVA package with default parameters:   - normalize = TRUE - alpha=0.25 - checkNA=’auto’ - use="everything" |
| JASMINE | ✓ | ✓ | Ranks genes per sample after removing zero-count genes to address dropout effects; computes a standardized pathway activity score (V statistic) averaged with the standardized odds ratio or likelihood function. | FUNCellA implementation; function gene2path with default effect size correction parameter:   - type="oddsratio" |
| Mean | ✓ | ✓ | Mean value of non-zero gene expressions for genes in the pathway. | FUNCellA implementation; function gene2path (no parameters). |
| Z-Score | X | ✓ | z-score normalisation performed across the genes; final score obtained via Stouffer’s integration. | FUNCellA implementation; function gene2path (no parameters). |
| BINA | ✓ | X | Drop-ratio as the fraction of non-zero genes in the pathway per sample (dropout scaled by pathway size); final score obtained via logit transformation. | FUNCellA implementation; function gene2path with default parameter:   - offset=0.1 |

**Supplementary Table 2. Detail summary of used ATD methods.**

| **Method** | **Previously published** | **Method description** | **Default run** |
| --- | --- | --- | --- |
| AUCell thresholding | ✓ | Choice of the threshold based on multiple approaches. | Function *auc_assignmnetThreshold_v6* from AUCell package with default parameters. |
| K-means thresholding | ✗ | Clustering of PAS using k-means algorithm. The samples belonging to the cluster with the highest mean is chosen as enriched. | FUNCellA implementation; function thr_KM with parameter:   - K=15 |
| GMM Top1 | ✗ | Clustering of PAS using GMM algorithm. The samples belonging to component with the highest mean is chosen as enriched. | FUNCellA implementation; function GMMdecomp with parameters:   - K=15 - IC=”AIC” - multiply=T   Next, function thr_GMM was run and Top1 threshold was extracted (no parameters). |
| GMM with k-means | ✗ | Clustering of PAS using GMM algorithm. Then, grouping the cluster parameters, the clusters are combined. The samples belonging to the group with the highest means is chosen as enriched. | FUNCellA implementation; function GMMdecomp with parameters:   - K=15 - IC=”AIC” - multiply=T   Next, function thr_GMM was run and k-means threshold was extracted (no parameters). |

**Supplementary Table 3. Detailed summary of used datasets.**

| **Dataset** | **Access no.** | **Set type** | **Cell Type** | **# of cells** | **Targeted** | **# of cells targeted** | **Total number of cells** | **Total number of transcripts** |
| --- | --- | --- | --- | --- | --- | --- | --- | --- |
| PBMC | GEO Series GSE132044 | Model dataset | Cytotoxic T cell | 1 174 | T cell | 1 724 | 3 222 | 15 817 |
|  |  |  | CD4+ T cell | 550 |  |  |  |  |
|  |  |  | B cell | 288 | B cell | 288 |  |  |
|  |  |  | Megakaryocyte | 221 | no pathways for targeting | |  |  |
|  |  |  | Natural killer cell | 166 | Natural killer cell | 166 |  |  |
|  |  |  | CD14+ monocyte | 640 | Monocyte | 742 |  |  |
|  |  |  | CD16+ monocyte | 102 |  |  |  |  |
|  |  |  | Dendritic cell | 55 | Dendritic cell | 55 |  |  |
|  |  |  | Plasmacytoid dendritic cell | 26 | Plasmacytoid dendritic cell | 26 |  |  |
| Bone Marrow (BM) | ArrayExpress E-MTAB-9580 | Validation set | Hematopoietic multipotent progenitor cell | 1 335 | cells not dedicated for test | | 3 918 | 18 874 |
|  |  |  | Progenitor cell | 583 | cells not dedicated for test | |  |  |
|  |  |  | Precursor B cell | 282 | B cell | 684 |  |  |
|  |  |  | Pro-B cell | 215 |  |  |  |  |
|  |  |  | Mature B cell | 187 |  |  |  |  |
|  |  |  | Monocyte | 267 | Monocyte | 267 |  |  |
|  |  |  | Granulocyte | 260 | cells not dedicated for test | |  |  |
|  |  |  | Mast cell | 186 | cells not dedicated for test | |  |  |
|  |  |  | Granulocyte monocyte progenitor cell | 174 | cells not dedicated for test | |  |  |
|  |  |  | Dendritic cell | 167 | Dendritic cell | 167 |  |  |
|  |  |  | Megakaryocyte | 92 | cells not dedicated for test | |  |  |
|  |  |  | Erythroid progenitor cell | 90 | cells not dedicated for test | |  |  |
|  |  |  | Natural killer cell | 42 | Natural killer cell | 42 |  |  |
|  |  |  | Endothelial cell | 38 | cells not dedicated for test | |  |  |
| Liver | NPC GSE159977 | Validation set | T cell | 7 676 | T cell | 7676 | 14 852 | 15 573 |
|  |  |  | Endothelial cell of hepatic sinusoid | 2 285 | cells not dedicated for test | |  |  |
|  |  |  | Macrophage | 1 625 | cells not dedicated for test | |  |  |
|  |  |  | Natural killer cell | 919 | Natural killer cell | 919 |  |  |
|  |  |  | Hepatocyte | 746 | cells not dedicated for test | |  |  |
|  |  |  | Kupffer cell | 619 | cells not dedicated for test | |  |  |
|  |  |  | B cell | 405 | B cell | 405 |  |  |
|  |  |  | Cholangiocyte | 292 | cells not dedicated for test | |  |  |
|  |  |  | Plasma cell | 162 | cells not dedicated for test | |  |  |
|  |  |  | Cycling cell | 57 | cells not dedicated for test | |  |  |
|  |  |  | Endothelial cell of vascular tree | 39 | cells not dedicated for test | |  |  |
|  |  |  | Hematopoietic stem cell | 27 | cells not dedicated for test | |  |  |
| COVID | ArrayExpress E-MTAB-9221 | Validation set | T cell | 2 546 | T cell | 2546 | 4 903 | 15 390 |
|  |  |  | Neutrophil | 770 | cells not dedicated for test | |  |  |
|  |  |  | Erythroid lineage cell | 485 | cells not dedicated for test | |  |  |
|  |  |  | Monocyte | 458 | Monocyte | 458 |  |  |
|  |  |  | B cell | 385 | B cell | 385 |  |  |
|  |  |  | Platelet | 259 | cells not dedicated for test | |  |  |

**Supplementary Table 4 List of used pathways with information about target cell type.**

| **ID** | **Title** | **Database** | **Target cell type** |
| --- | --- | --- | --- |
| hsa04660 | T cell receptor signaling pathway | KEGG | T cell |
| hsa04662 | B cell receptor signaling pathway | KEGG | B cell |
| CM00003 | B cell | CellMarker | B cell |
| CM00009 | CD4+ T cell | CellMarker | T cell |
| CM00010 | CD8+ T cell | CellMarker | T cell |
| CM00011 | Dendritic cell | CellMarker | Dendritic Cell |
| CM00028 | Monocyte | CellMarker | Monocyte |
| CM00032 | Natural killer cell | CellMarker | Natural Killer |
| CM00038 | Plasmacytoid dendritic cell | CellMarker | Plasmacytoid dendritic cell |
| CM00042 | Regulatory T (Treg) cell | CellMarker | T cell |
| CM00046 | T cell | CellMarker | T cell |
| CM00047 | T helper cell | CellMarker | T cell |
| CS00001 | B cells memory top10 | CIBERSORT | B cell |
| CS00002 | B cells memory top25 | CIBERSORT | B cell |
| CS00003 | B cells memory top50 | CIBERSORT | B cell |
| CS00004 | B cells naive top10 | CIBERSORT | B cell |
| CS00005 | B cells naive top25 | CIBERSORT | B cell |
| CS00006 | B cells naive top50 | CIBERSORT | B cell |
| CS00007 | Dendritic cells activated top10 | CIBERSORT | Dendritic Cell |
| CS00008 | Dendritic cells activated top25 | CIBERSORT | Dendritic Cell |
| CS00009 | Dendritic cells activated top50 | CIBERSORT | Dendritic Cell |
| CS00010 | Dendritic cells resting top10 | CIBERSORT | Dendritic Cell |
| CS00011 | Dendritic cells resting top25 | CIBERSORT | Dendritic Cell |
| CS00012 | Dendritic cells resting top50 | CIBERSORT | Dendritic Cell |
| CS00031 | Monocytes top10 | CIBERSORT | Monocyte |
| CS00032 | Monocytes top25 | CIBERSORT | Monocyte |
| CS00033 | Monocytes top50 | CIBERSORT | Monocyte |
| CS00037 | NK cells activated top10 | CIBERSORT | Natural Killer |
| CS00038 | NK cells activated top25 | CIBERSORT | Natural Killer |
| CS00039 | NK cells activated top50 | CIBERSORT | Natural Killer |
| CS00040 | NK cells resting top10 | CIBERSORT | Natural Killer |
| CS00041 | NK cells resting top25 | CIBERSORT | Natural Killer |
| CS00042 | NK cells resting top50 | CIBERSORT | Natural Killer |
| CS00046 | T cells CD4 memory activated top10 | CIBERSORT | T cell |
| CS00047 | T cells CD4 memory activated top25 | CIBERSORT | T cell |
| CS00048 | T cells CD4 memory activated top50 | CIBERSORT | T cell |
| CS00049 | T cells CD4 memory resting top10 | CIBERSORT | T cell |
| CS00050 | T cells CD4 memory resting top25 | CIBERSORT | T cell |
| CS00051 | T cells CD4 memory resting top50 | CIBERSORT | T cell |
| CS00052 | T cells CD4 naive top10 | CIBERSORT | T cell |
| CS00053 | T cells CD4 naive top25 | CIBERSORT | T cell |
| CS00054 | T cells CD4 naive top50 | CIBERSORT | T cell |
| CS00055 | T cells CD8 top10 | CIBERSORT | T cell |
| CS00056 | T cells CD8 top25 | CIBERSORT | T cell |
| CS00057 | T cells CD8 top50 | CIBERSORT | T cell |
| CS00058 | T cells follicular helper top10 | CIBERSORT | T cell |
| CS00059 | T cells follicular helper top25 | CIBERSORT | T cell |
| CS00060 | T cells follicular helper top50 | CIBERSORT | T cell |
| CS00061 | T cells gamma delta top10 | CIBERSORT | T cell |
| CS00062 | T cells gamma delta top25 | CIBERSORT | T cell |
| CS00063 | T cells gamma delta top50 | CIBERSORT | T cell |
| CS00064 | T cells regulatory (Tregs) top10 | CIBERSORT | T cell |
| CS00065 | T cells regulatory (Tregs) top25 | CIBERSORT | T cell |
| CS00066 | T cells regulatory (Tregs) top50 | CIBERSORT | T cell |
| PG00009 | B cell | PanglaoDB | B cell |
| PG00010 | B cells memory | PanglaoDB | B cell |
| PG00011 | B cells naive | PanglaoDB | B cell |
| PG00028 | Dendritic cell | PanglaoDB | Dendritic Cell |
| PG00053 | Gamma delta T cell | PanglaoDB | T cell |
| PG00086 | Monocytes | PanglaoDB | Monocyte |
| PG00093 | Natural killer T cell | PanglaoDB | Natural Killer |
| PG00099 | NK cell | PanglaoDB | Natural Killer |
| PG00116 | Plasmacytoid dendritic cell | PanglaoDB | Plasmacytoid dendritic cell |
| PG00139 | T cell | PanglaoDB | T cell |
| PG00140 | T follicular helper cell | PanglaoDB | T cell |
| PG00141 | T helper cell | PanglaoDB | T cell |
| PG00142 | T memory cell | PanglaoDB | T cell |
| PG00143 | T regulatory cell | PanglaoDB | T cell |
| PG00146 | Thymocytes | PanglaoDB | T cell |
| PG00147 | Transient cell | PanglaoDB | T cell |
| LI.M4.5 | mitotic cell cycle in stimulated CD4 T cells | tmod | T cell |
| LI.M4.6 | cell division in stimulated CD4 T cells | tmod | T cell |
| LI.M4.9 | mitotic cell cycle in stimulated CD4 T cells | tmod | T cell |
| LI.M4.11 | mitotic cell cycle in stimulated CD4 T cells | tmod | T cell |
| LI.M4.15 | enriched in monocytes (I) | tmod | Monocyte |
| LI.M5.1 | T cell activation and signaling | tmod | T cell |
| LI.M7.0 | enriched in T cells (I) | tmod | T cell |
| LI.M7.1 | T cell activation (I) | tmod | T cell |
| LI.M7.2 | enriched in NK cells (I) | tmod | Natural Killer |
| LI.M7.3 | T cell activation (II) | tmod | T cell |
| LI.M7.4 | T cell activation (III) | tmod | T cell |
| LI.M9 | B cell development | tmod | B cell |
| LI.M11.0 | enriched in monocytes (II) | tmod | Monocyte |
| LI.M14 | T cell differentiation | tmod | T cell |
| LI.M18 | T cell differentiation via ITK and PKC | tmod | T cell |
| LI.M19 | T cell differentiation (Th2) | tmod | T cell |
| LI.M23 | RA, WNT, CSF receptors network (monocyte) | tmod | Monocyte |
| LI.M35.0 | signaling in T cells (I) | tmod | T cell |
| LI.M35.1 | signaling in T cells (II) | tmod | T cell |
| LI.M36 | T cell surface, activation | tmod | T cell |
| LI.M40 | complement and other receptors in DCs | tmod | Dendritic Cell |
| LI.M43.0 | myeloid, dendritic cell activation via NFkB (I) | tmod | Dendritic Cell |
| LI.M43.1 | myeloid, dendritic cell activation via NFkB (II) | tmod | Dendritic Cell |
| LI.M44 | T cell signaling and costimulation | tmod | T cell |
| LI.M46 | cell division stimulated CD4+ T cells | tmod | T cell |
| LI.M47.0 | enriched in B cells (I) | tmod | B cell |
| LI.M47.1 | enriched in B cells (II) | tmod | B cell |
| LI.M47.2 | enriched in B cells (III) | tmod | B cell |
| LI.M47.3 | enriched in B cells (IV) | tmod | B cell |
| LI.M47.4 | enriched in B cells (V) | tmod | B cell |
| LI.M50 | CD1 and other DC receptors | tmod | Dendritic Cell |
| LI.M52 | T cell activation (IV) | tmod | T cell |
| LI.M57 | immuregulation - monocytes, T and B cells | tmod | Monocyte |
| LI.M58 | B cell development/activation | tmod | B cell |
| LI.M61.2 | enriched in NK cells (receptor activation) | tmod | Natural Killer |
| LI.M64 | enriched in activated dendritic cells/monocytes | tmod | Dendritic Cell |
| LI.M67 | activated dendritic cells | tmod | Dendritic Cell |
| LI.M69 | enriched in B cells (VI) | tmod | B cell |
| LI.M73 | enriched in monocytes (III) | tmod | Monocyte |
| LI.M81 | enriched in myeloid cells and monocytes | tmod | Monocyte |
| LI.M83 | enriched in naive and memory B cells | tmod | B cell |
| LI.M86.1 | proinflammatory dendritic cell, myeloid cell response | tmod | Dendritic Cell |
| LI.M118.0 | enriched in monocytes (IV) | tmod | Monocyte |
| LI.M118.1 | enriched in monocytes (surface) | tmod | Monocyte |
| LI.M119 | enriched in activated dendritic cells (I) | tmod | Dendritic Cell |
| LI.M123 | enriched in B cell differentiation | tmod | B cell |
| LI.M157 | enriched in NK cells (III) | tmod | Natural Killer |
| LI.M165 | enriched in activated dendritic cells (II) | tmod | Dendritic Cell |
| LI.M168 | enriched in dendritic cells | tmod | Dendritic Cell |
| LI.M223 | enriched in T cells (II) | tmod | T cell |
| LI.S0 | T cell surface signature | tmod | T cell |
| LI.S1 | NK cell surface signature | tmod | Natural Killer |
| LI.S4 | Monocyte surface signature | tmod | Monocyte |
| LI.S5 | DC surface signature | tmod | Dendritic Cell |
| LI.S6 | CD4 T cell surface signature Th1-stimulated | tmod | T cell |
| LI.S7 | CD4 T cell surface signature Th2-stimulated | tmod | T cell |
| LI.S10 | Resting dendritic cell surface signature | tmod | Dendritic Cell |
| LI.S11 | Activated (LPS) dendritic cell surface signature | tmod | Dendritic Cell |
| DC.M3.6 | Cytotoxic/NK Cell | tmod | Natural Killer |
| DC.M4.1 | T cell | tmod | T cell |
| DC.M4.10 | B cell | tmod | B cell |
| DC.M4.14 | Monocytes | tmod | Monocyte |
| DC.M4.15 | T cells | tmod | T cell |
| DC.M8.46 | Cytotoxic/NK | tmod | Natural Killer |

**Supplementary Table 5 Summary of used pathways per target cell type and database.**

| **Cell type** | **KEGG** | **CellMarker** | **CYBER-SORT** | **tmod** | **PangolaDB** |
| --- | --- | --- | --- | --- | --- |
| B cell | 1 | 1 | 6 | 11 | 3 |
| T cell | 1 | 5 | 21 | 24 | 8 |
| Natural Killer | 0 | 1 | 6 | 6 | 2 |
| Dendritic Cell | 0 | 1 | 6 | 13 | 1 |
| Monocytes | 0 | 1 | 3 | 10 | 1 |
| Plasmoid dendritic cell | 0 | 1 | 0 | 0 | 1 |
| Total | 2 | 10 | 42 | 64 | 16 |

**Supplementary Table 6 List of signature genes for HER2+ breast cancer subtype and GAMs cells.**

| **HER2+ signature genes** | **GAMs signature genes** |
| --- | --- |
| ABCA12 | C3 |
| C12orf28 | MEF2C |
| C7orf24 | A2M |
| CRKRS | CX3CR1 |
| FA2H | NAV3 |
| FHOD1 | GPR34 |
| FNTA | POU2F2 |
| GRB7 | PLXDC2 |
| GSDML | TREM2 |
| GSR | APOC2 |
| HDAC5 | MIF |
| KATNAL2 KMO | S100A10 |
| MED1 | LGALS1 |
| PMAIP1 |  |
| PNMT |  |
| PSMD3 |  |
| SAMD11 |  |
| STARD3 |  |
| TCAP |  |
| TP53INP2 |  |

**Supplementary Table 7 List of analysed pathways per dataset and enrichment method.**

| **Dataset \ enrichment method** | **AUCell** | **CERNO** | **ssGSEA** | **JASMINE** | **Mean** | **Z-Score** | **BINA** |
| --- | --- | --- | --- | --- | --- | --- | --- |
| **PBMC [n=3 222] - model train set** | 21 | 33 | 29 | 20 | 34 | 29 | 25 |
| **LIVER [n=14 852] - validation** | 12 | 19 | 16 | 12 | 17 | 14 | 12 |
| **BM [n=3 918] - validation** | 19 | 24 | 23 | 20 | 26 | 23 | 21 |
| **COVID [n=4 903] - validation** | 17 | 28 | 24 | 18 | 28 | 23 | 18 |
| **Total** | **69** | **104** | **92** | **70** | **105** | **89** | **76** |
| **Total - validation** | **48** | **71** | **63** | **50** | **71** | **60** | **51** |

**Supplementary Table 8 Results of statistical analysis for comparison ssEA between each other.**

| **ARI** | **P-values for hypothesis that the algorithm in column performs better than the one in row.** | | | | | | | | |
| --- | --- | --- | --- | --- | --- | --- | --- | --- | --- |
|  | **AUCell - GMM with KM** | **CERNO - GMM with K-M** | **CERNO - K-M thresholding** | **ssGSEA - GMM with K-M** | **JASMINE - GMM with K-M** | **JASMINE - K-M thresholding** | **Mean - K-M thresholding** | **Z-Score - K-M thresholding** | **BINA - GMM with K-M** |
| **AUCell - GMM with KM** | *NA* | 0.6390 | 0.1954 | 0.8398 | 1.0000 | 1.0000 | 0.1334 | 0.1668 | 1.0000 |
| **CERNO - GMM with K-M** | 0.3610 | *NA* | 0.1469 | 0.6937 | 1.0000 | 1.0000 | 0.0571 | 0.1348 | 1.0000 |
| **CERNO - K-M thresholding** | 0.8046 | 0.8531 | *NA* | 0.9470 | 1.0000 | 1.0000 | 0.3667 | 0.4705 | 1.0000 |
| **ssGSEA - GMM with K-M** | 0.1602 | 0.3063 | 0.0530 | *NA* | 1.0000 | 1.0000 | **0.0267** | **0.0385** | 1.0000 |
| **JASMINE - GMM with K-M** | **0.0000** | **0.0000** | **0.0000** | **0.0000** | *NA* | 0.0885 | **0.0000** | **0.0000** | **0.0040** |
| **JASMINE - K-M thresholding** | **0.0000** | **0.0000** | **0.0000** | **0.0000** | 0.9115 | *NA* | **0.0000** | **0.0000** | **0.0446** |
| **Mean - K-M thresholding** | 0.8666 | 0.9429 | 0.6333 | 0.9733 | 1.0000 | 1.0000 | *NA* | 0.6403 | 1.0000 |
| **Z-Score - K-M thresholding** | 0.8332 | 0.8652 | 0.5295 | 0.9615 | 1.0000 | 1.0000 | 0.3597 | *NA* | 1.0000 |
| **BINA - GMM with K-M** | **0.0000** | **0.0000** | **0.0000** | **0.0000** | 0.9960 | 0.9554 | **0.0000** | **0.0000** | *NA* |
| **FDR** | **P-values for hypothesis that the algorithm in column performs better than the one in row.** | | | | | | | | |
|  | **AUCell - GMM with KM** | **CERNO - GMM with K-M** | **CERNO - K-M thresholding** | **ssGSEA - GMM with K-M** | **JASMINE - GMM with K-M** | **JASMINE - K-M thresholding** | **Mean - K-M thresholding** | **Z-Score - K-M thresholding** | **BINA - GMM with K-M** |
| **AUCell - GMM with KM** | *NA* | 0.4421 | 0.5318 | 0.7357 | 0.9517 | 1.0000 | **0.0207** | **0.0031** | 0.9999 |
| **CERNO - GMM with K-M** | 0.5579 | *NA* | 0.5927 | 0.8266 | 0.9507 | 1.0000 | **0.0174** | **0.0009** | 1.0000 |
| **CERNO - K-M thresholding** | 0.4682 | 0.4073 | *NA* | 0.6928 | 0.9473 | 1.0000 | **0.0103** | **0.0005** | 1.0000 |
| **ssGSEA - GMM with K-M** | 0.2643 | 0.1734 | 0.3072 | *NA* | 0.9035 | 1.0000 | **0.0004** | **0.0001** | 1.0000 |
| **JASMINE - GMM with K-M** | **0.0483** | **0.0493** | 0.0527 | 0.0965 | *NA* | 0.9995 | **0.0006** | **0.0006** | 0.9596 |
| **JASMINE - K-M thresholding** | **0.0000** | **0.0000** | **0.0000** | **0.0000** | **0.0005** | ***NA*** | **0.0000** | **0.0000** | **0.0462** |
| **Mean - K-M thresholding** | 0.9793 | 0.9826 | 0.9897 | 0.9996 | 0.9994 | 1.0000 | *NA* | 0.4190 | 1.0000 |
| **Z-Score - K-M thresholding** | 0.9969 | 0.9991 | 0.9995 | 0.9999 | 0.9994 | 1.0000 | 0.5810 | *NA* | 1.0000 |
| **BINA - GMM with K-M** | **0.0001** | **0.0000** | **0.0000** | **0.0000** | **0.0404** | 0.9538 | **0.0000** | **0.0000** | *NA* |
